# Recent Advances in Immunosafety and Nanoinformatics of Two-Dimensional Materials Applied to Nano-imaging

**DOI:** 10.3389/fimmu.2021.689519

**Published:** 2021-06-03

**Authors:** Gabriela H. Da Silva, Lidiane S. Franqui, Romana Petry, Marcella T. Maia, Leandro C. Fonseca, Adalberto Fazzio, Oswaldo L. Alves, Diego Stéfani T. Martinez

**Affiliations:** ^1^ Brazilian Nanotechnology National Laboratory (LNNano), Brazilian Center for Research in Energy and Materials (CNPEM), Campinas, Brazil; ^2^ School of Technology, University of Campinas (Unicamp), Limeira, Brazil; ^3^ Center of Natural and Human Sciences, Federal University of ABC (UFABC), Santo Andre, Brazil; ^4^ NanoBioss Laboratory and Solid State Chemistry Laboratory (LQES), Institute of Chemistry, University of Campinas (Unicamp), Campinas, Brazil

**Keywords:** nanomaterials, bioimaging, immunotoxicity, nanobiotechnology, nanosafety

## Abstract

Two-dimensional (2D) materials have emerged as an important class of nanomaterials for technological innovation due to their remarkable physicochemical properties, including sheet-like morphology and minimal thickness, high surface area, tuneable chemical composition, and surface functionalization. These materials are being proposed for new applications in energy, health, and the environment; these are all strategic society sectors toward sustainable development. Specifically, 2D materials for nano-imaging have shown exciting opportunities in *in vitro* and *in vivo* models, providing novel molecular imaging techniques such as computed tomography, magnetic resonance imaging, fluorescence and luminescence optical imaging and others. Therefore, given the growing interest in 2D materials, it is mandatory to evaluate their impact on the immune system in a broader sense, because it is responsible for detecting and eliminating foreign agents in living organisms. This mini-review presents an overview on the frontier of research involving 2D materials applications, nano-imaging and their immunosafety aspects. Finally, we highlight the importance of nanoinformatics approaches and computational modeling for a deeper understanding of the links between nanomaterial physicochemical properties and biological responses (immunotoxicity/biocompatibility) towards enabling immunosafety-by-design 2D materials.

## Introduction

Two-dimensional (2D) materials constitutes an emerging class of nanomaterials, characterized mainly by their high surface-area-to-mass ratio due to a sheet-like morphology; responsible for their outstanding physicochemical properties (e.g., electronic, optical, mechanical, and magnetic) with a currently leading position in materials science and technology (1, 2). Since the pioneering work of Novoselov et al. (3) in 2004, several 2D materials have been produced for many applications in energy, catalysis, composites, sensors, biomedicine, agriculture, and environmental remmediation (4–7).

Beyond graphene-based materials (GBMs), other 2D materials have also emerged, by replacing carbon elements for other heteroatoms (P, B, O, and N) (8). Black phosphorus (BP), transition metal dichalcogenides (TMDs), transition metal carbides, nitrides, and carbonitrides (MXenes), layered double hydroxides (LDHs), antimonenes (AM), boron nitride nanosheets (BNNs) are the most common graphene analogs under investigation (9–17).

Among several applications, 2D materials have attracted special interest to be applied in the bioimaging field because of their high electrical and thermal conductivity, high degree of anisotropy, exceptional mechanical strength, and unique optical properties (18). Due to such properties, 2D materials have been developed to be applied in molecular imaging techniques, such as computed tomography (CT), magnetic resonance imaging (MRI), optical imaging (fluorescence and luminescence), and nuclear imaging including positron emission tomography (PET) and single photon emission computed tomography (SPECT) (19). Besides, 2D materials allow multimodal imaging by providing a variety of properties useful for more than one imaging technique and/or because of their facility to combine them to form nanocomposites and hybrid materials (20). Given the applicability and growing interests in 2D materials, unveiling their impact on the immune system is a key step towards safe use and responsible innovation (21, 22). These materials’ intrinsic characteristics, such as chemical composition, surface chemistry, functionalization, morphology, lateral size, purity, and crystallinity are directed related to their degradability, dispersion stability, and protein corona profile; hence, their adverse effects in a biological system (23–26). Such parameters modulate the biotransformation and biodistribution of 2D materials under *in vitro* and *in vivo* models, influencing their interaction with the immune system, fate, and toxicological profile (27–30).

Biocompatibility, biodegradability, and eliciting an adequate biological effect in the organisms are crucial to the applicability of 2D materials (22, 24, 31). Indeed, the complexity of toxicokinetic and toxicodynamic events of 2D materials under physiological conditions associated with a lack of harmonized protocols for experimental research represents majors challenges for clinical translation and safety regulation involving these emerging materials (32–35). Therefore, combining systems toxicology and nanoinformatics is a foremost strategy in the integration of 2D material design on a safe and sustainable basis (36–38).

**Figure 1:**
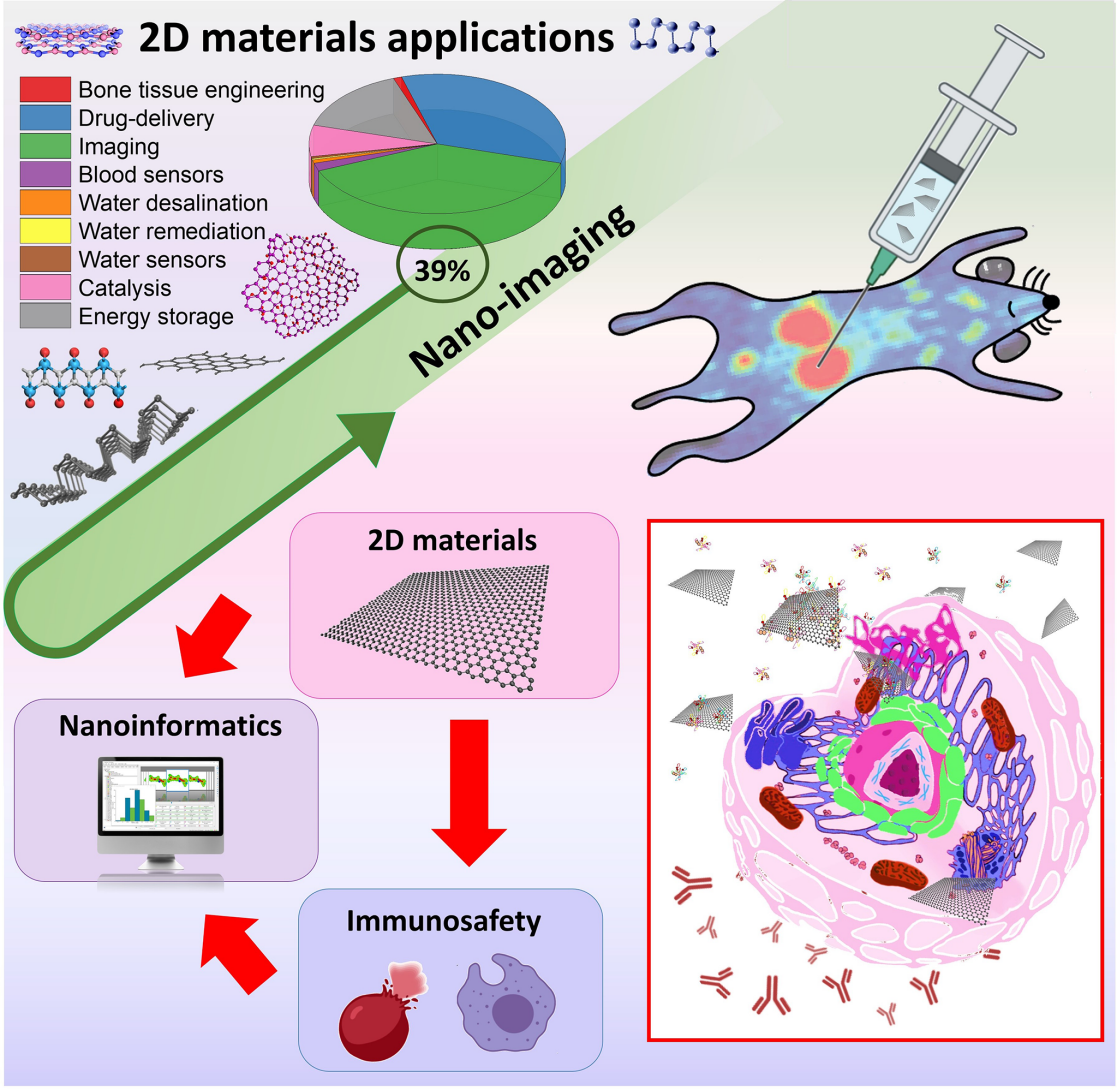
Two-dimensional materials applications, nano-imaging and their links with immunosafety and nanoinformatics approaches.

In this mini-review, we present the recent advances involving 2D materials, nano-imaging, and immunosafety. Briefly, the main findings associated with the adverse immunological effects were shown in *in vitro* and *in vivo* models. Finally, we highlight the great potential of nanoinformatics approaches towards immunosafety-by-design 2D materials (**Figure 1**).

## Technological Applications And Innovation Of 2d Materials

**Figure 2:**
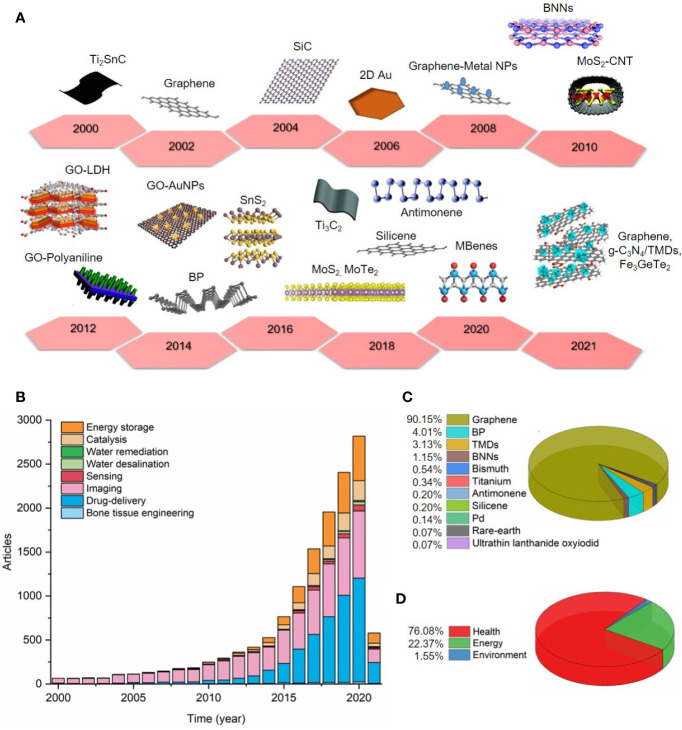
The data obtained previously was organized into the following sectors: health (bone tissue engineering, drug delivery, imaging, sensing blood markers), energy (catalysis and energy storage), and environment (water remediation and desalination, and water sensing contaminants). **(A)** Timeline showing examples of 2D materials produced over the period established (from 2000 to 2021). **(B)** Number of articles from 2000 to 2021 (25/03/2021) **(C)** 2D materials used in nano-imaging applications (see supporting information) **(D)** Percentage of 2D materials applied in health, energy and environment sectors.

A literature review on the Web of Science™ database was performed, considering articles published from 2000 to 2021 (25/03/2021), and over these last 20 years, many 2D materials have been synthesized as exemplified in **Figure 2A**. The number of publications of 2D materials and their applications is growing, in which nano-imaging and drug release systems stand out and are present mostly in the health sector (**Figures 2B, D** **)**. For energy application, the structural and electronic properties of 2D materials have been shown to improve the energy accumulation in devices such as lithium-ion, metal-air batteries (LIBs) (9, 39, 49, 50) and electrochemical devices (51, 52). Moreover, these 2D materials are of particular interest as catalysts and nanoscale substrates, replacing transition, or noble metals normally used to catalyze an acid-basic reaction, producing metal free-catalysts (53, 54). In environment, the 2D materials have been used as adsorbents for removing pollutants to treat contaminated water (55–57). Their atomic thickness and antibacterial activity contribute to superior water permeability and anti-fouling capacity in the development of membranes for desalination (58–62) and cleaning purposes (63–65). Sensing has covered both environmental and health sectors, contributing to the detection and monitoring of traces of pollutants (66, 67) and blood biomarkers (68–71). The thin structure, large surface area, chemical modifications and quenching ability of 2D materials provide high sensitivity, durability, stability, selectivity, and conductivity for sensors and biosensors (72–82).

Considering biomedical applications, 2D materials have been applied in bone tissue engineering, conferring improved mechanical characteristics and great osteoconductivity for scaffold design (83–87). However, due to the higher surface area of 2D materials and distinguish light-material interactions, research has mostly given attention to their usefulness in nano-imaging and therapeutics (theranostics) (88) (**Figures 2B, C**), including early detection, monitoring, and treatment of diseases, which are the main examples described in this mini-review (89). For example, in cancer, malign tumors are sensitive to heating when compared to healthy tissues. Graphene oxide decorated with gold nanoparticles (GO-AuNPs), TMDs (MoS_2_, WS_2_), and MXenes (MoC_2_, Ti_3_C_2_) have shown effective agents in photothermal and photodynamic therapy for inducing tumor necrosis (40, 41, 90–92). 2D materials have been successfully modified with numerous polymers to enhance their cytocompatibility and dispersibility (90) and used as nanoplatforms carrying active molecules or imaging agents to improve their biological function (93) and clinical visualization for imaging-drug delivery guiding (12, 94). MoS_2_ and BNNs have been employed as effective fluorescence quenchers and associated with aptamers, substituting antibody-based therapy (69, 95–97). Compared to the other 2D inorganic materials, and in addition to the previous features, the ultrathin structure of the BP nanosheets results in an exceptional biodegradability in physiological media it shows promising in theranostics (98, 99). Magnetic nanoparticles have been used as contrast agents and incorporated into 2D materials in MRI, in place of conventional ones (100, 101). In this respect, 2D magnetic materials production can be very useful for accurate bioimaging and therapy of diseases *in vivo* using MRI and CT techniques (10, 102).

## 2D Materials And The Immune System: Adverse Effects In *In Vitro* And *In Vivo* Models

As far as it is known, 2D materials have proven their significance and innovation perspective in almost all industrial areas and sectors, making it imperative to assess their environmental health risks and safety aspects (24, 103–105). However, toxicological studies, including immunotoxicity, are still in their infancy for GBMs and 2D inorganic materials (31). **Table 1** is an extensive literature revision reporting major findings of 2D materials and their adverse effects in the immunological system considering *in vitro* and *in vivo* models. The terms used for the literature research is detailed in the supplementary material.

**Table 1:** Relevant studies addressing the adverse immunological effects of 2D materials in *in vitro* and *in vivo* models from 2000 to 2021.

Nanomaterial	Dose	Exposure time	*in vivo*/*in vitro* models	Method or endpoints	Adverse immunological effects	Ref.
Graphene oxide (GO) (lateral size of 350 nm and 2 µm)	2, 4, and 6 µg ml^−1^	24, 48, and 96 h	peritoneal macrophage	Secretion of pro-inflammatory cytokines (IL-6, IL-10, IL-12, TNF-α, MCP-1, and IFN-ɤ)	Dose-dependent release of cytokines induced in a higher extent by 2 µm GO than 350 nm GO.	(106)
	–	21 days	C57BL/6 male mice	Histological micrographics	Mononuclear cells (i.e. macrophages and lymphocytes) infiltration and inflammation response induced by 2 µm GO, but not by 350 nm GO.
GO (smallest S-GO 50–350 nm; intermediate I-GO 350–750 nm; largest L-GO 750–1300 nm)	Viability: 1-300 µg ml^−1^; Others: 20 µg ml^−1^	12, 24 h	J774.A1 and THP-1 macrophages	Live/dead assay, TNF-α, IL-6 and IL1β release; and macrophage polarization, NF-κB signaling activation.	All GO materials have induced a decrease in cell viability, and a production of cytokines. The L-GO significantly elicited higher response than S-GO. Higher macrophage polarization to the M1 phenotype by L-GO than S-GO.	(107)
	*Ip^1^:* 5000 μg kg^−1^ bw; Lung^2^: 2500 μg kg^−1^ bw; It^3^: 5000 μg kg^−1^ bw	Ip: 72 h; Lung: 72 h; It: 24 h	BALB/c male mice	Local and systemic inflammation: TNF-α, IL6 release, recruitment of immune cell.	Both S-GO and L-GO have induced an inflammatory response by cytokines production and leukocytes recruitment, been the L-GO response higher than the S-GO response in all endpoints.
GO S-GO (<1 µm) L-GO (1-10 µM)	25, 50, and 75 µg ml^−1^	24 h	PBMCs, Jurkat and THP-1 cells	Annexin-V FITC (apoptosis), LIVE/DEAD FITC (late apoptosis and necrosis), and propidium iodide (necrosis), cell activation (expression of CD69 and CD25 markers), cytokine release, expression of 84 genes related to innate and adaptive immune responses	Only S-GO presented a decrease in cell viability at highest dose (75 µg/ml). None of GO tested have induced the cell activation (expression of CD69 and CD25 markers). However, both GO induced cytokines release and upregulation of genes related to immune response, being that the S-GO response was significantly higher compared to L-GO response.	(108)
GO-PEG (200-500 nm) and PG-FMN (L) (200-400 nm) and PG-FMN (S) (100-200 nm)	10 μg ml^−1^	24 h	RAW-264.7 macrophages	Cellular uptake, nitric oxide production, NMR metabolic profiling, expression of cell surface markers CD80 and CD206.	PG-FMN (S) was internalized in a greater extent compared to GO-PEG and PG-FMN (L), which presented a similar uptake. GO-PEG did not induce NO production, whereas PG-FMN (S) and PG-FMN (L) caused significant NO increases of 21% and 12%, respectively. Only PG-FMN (S) caused increases in intracellular succinate and itaconate, similarly to LPS, while PG-FMN (L) did not alter the levels of TCA cycle intermediates and GO-PEG caused a decrease of succinate. Besides, GO-PEG decreased the TNF-α secretion compared to control cell, and do not affected the cell surface markers.	(109)
GO-PEG (200-500 nm)	40 and 80 μg ml^−1^	24 and 48 h	Murine peritoneal macrophages	Cell surface markers of M1 (CD80 and iNOS) and M2 (CD206 and CD163) phenotypes.	PEG-GO did not induce the macrophage polarization towards the M1 pro-inflammatory phenotype, with a slight shift towards M2 reparative phenotype.	(110)
GO-1PEG (~100 nm) GO-6PEG (~300 nm)	2.3–75 µg ml^−1^	24 h	RAW-264.7 macrophages and primary splenocytes (B-cells and T-cells)	Proinflammatory cytokine secretion (IL-1β, TNF-α and IL-6) and proliferation of immune cells.	Only GO-6PEG increased the secretion of TNF-α by RAW-264.7 macrophages without alteration of IL-6 and IL-1β levels. The treatment of primary splenocytes with GO-1PEG and GO-6PEG in the presence of concanavalin A, anti-CD3 antibody, and LPS, produced significant dose-dependent decrease of cell proliferation and IL-6 levels.	(111)
GO and PVP coated-GO	25, 50, and 100 µg ml^−1^	48 h	Human DC, macrophages and T cells	Differentiation and maturation of DC cells, cytokine release, apoptosis of T cells, and phagocytosis	GO induced the differentiation and maturation of DC cells; a dose-dependent release of pro-inflammatory cytokines by DC cells; a dose-dependent apoptosis of T cells; and a susceptibility of phagocytosis by macrophages. The coating with PVP has reduced the cytokines secretion and the differentiation and maturation of DC cells; delayed the apoptotic process of T cells; and avoid the phagocytosis by macrophages.	(112)
GO GO-NH_2,_ GO-PAM, GO-PAA GO-PEG	1, 2, 4, 10, 20, 50, 100, or 200 μg ml^−1^	1, 6, and 24 h	J774A.1 cell line	Viability, cellular adhesion, uptake, membrane permeability and fluidity, Ca^2+^ flux and transcriptome analysis.	GO caused the impairment of cell membrane integrity and functions including regulation of membrane- and cytoskeleton- associated genes, membrane permeability, fluidity, and ion channels. The -NH_2_ and -PAA showed similar toxicity to GO, but -PEG and -PAA significantly decreased the GO cytotoxicity.	(113)
	It: 1 mg kg^−1^	24 h	Male BALB/c mice	Survival, body weight increase, complete blood count (numbers of RBC, WBC, PLT, neutrophils, lymphocyte), blood biochemistry, GO distribution, histological analysis of lung, liver and spleen.	GO induced platelet depletion, pro-inflammatory response and pathological changes of lung and liver in mice. The -NH_2_, -PAA and -PEG modifications greatly reduced the toxicity of GO in mice. The -PAM modification was more toxic than pristine GO.	
GO and reduced GO (rGO) (100 nm)	20, 40, 60, 80, and 100 µg ml^−1^	24 h	THP-1 cells	Cellular viability, proliferation, oxidative stress, mitochondrial membrane potential, ATP synthesis, antioxidants, apoptosis, DNA damage, and the inflammation response	Both GO and rGO caused dose-dependent loss of cell viability and proliferation, increased level of LDH, MMP, decreased level of ATP content, redox imbalance, mitochondria-mediated apoptosis, cell death due to oxidative stress, increased secretion of various cytokines and chemokines. Overall, the toxic response of rGO was more severe than GO for all endpoints.	(114)
GO nanoplatelets (GONPs) and reduced GONPs (rGONPs)	GONP (5 µg ml^−1^) or rGONP (50 µg ml^−1^	24 h	THP-1 cells	Cell viability, ROS production, expression of genes related to the oxidative and inflammatory response, cellular uptake, endocytosis and phagocytosis, Rho/ROCK pathway, cytoskeleton analysis, differentiation of THP-1 cells into macrophage-like cells (THP-1a)	Both GO induced a dose-dependent loss in cell viability, an increase in ROS production, and a disruption of the F-actin cytoskeletons leading to the loss of the adherence ability of THP-1a and a reduction in the phagocytosis capability of THP-1a cells. GONP presented higher upregulation of HO-1 and SOD-2 expressions, and higher levels of IL-1β, TNF-α, IL-8, and MCP-1, compared to rGONP. rGONP exhibited a greater expression of NF-кB (p65), higher uptake and a higher decrease of Rho/ROCK expression than GONP.	(115)
Pristine graphene with 1% pluronic F108	20 µg ml^−1^	24 h	Primary and immortalize (RAW264.7) macrophages	Quantification of cytokines and chemokines (IFNɤ, IL-1α, IL-2, IL-4, IL-5, IL-6, IL-10, IL-17, TNFα, and GM-CSF, MCP-1, MCP-3, RANTES, MIP-1α and MIP-1β). RT-PCR analysis of the mRNA levels of TNF-α, IL-1β, IL-6, iNOS and COX-2. Adhesion, phagocytosis and cytoskeleton assay.	Increased transcription and secretion of cytokines and chemokines, which is triggered by activation of the NF-kB signaling pathway; The cytokines and chemokines secreted by graphene-exposed macrophages further impaired the morphology of naïve macrophages by affect the actin structures and podosomes expansion, decreasing the adhesion and phagocytosis.	(116)
Pristine graphene with 1% pluronic F108 (500–1000 nm)	20 µg ml^−1^	12, 24, and 48 h	Murine RAW 264.7 macrophages	Cell viability, ROS production, MMP, apoptosis, expression of proteins (Phospho-p38 MAPKinase (P-p38), p38 MAPKinase (p38), Phospho-JNK (P-JNK), JNK, Phospho-ERK (P-ERK), ERK, Phospho- Smad2, Smad2, Bim, Bax, caspase 3, Bcl-2, PARP and β-actin) and genes (TNF-α, TGF-β TGF-β receptor I, TGF-β receptor II, Smad2, Smad3, Smad4, Smad7, β-Actin)	Loss of cell viability at highest concentration (100 µg/mL); induction of intracellular ROS generation, depletion of MMP and apoptosis, all in a time- and dose-dependent way; activation of the mitochondrial pathways: MAPKs (JNK, ERK and p38) as well as the TGF- β-related signaling pathways.	(117)
Graphene nanoplatelets (1-10 layers)	1, 5 and 10 μg cm^2^	24 h	THP-1 macrophages	Phagocytosis, cytokine release and the involvement of the NALP3 inflammasome.	Frustrated phagocytosis, loss of membrane integrity at higher concentration, increase in cytokines expression, and activation of the NALP3 inflammasome.	(118)
	pharyngeal aspiration: 50 μg per mouse. intrapleural injection: 5 μg per mouse	24 h	C57BL/6 strain mice	BAL cells analysis, Histological sections of lungs. Pleural space lavage: total and differential cell count, histological examination of the parietal pleura.	BAL and pleural lavage showed an increased number of polymorphonuclear leucocytes (neutrophils and eosinophils); and an increase in the levels of cytokines. Histological analysis: presence of granulomatous lesions in the bronchiole lumen and near the alveolar region; presence of histiocytic aggregates along the mesothelium.	
Graphene nanoplatelets (~10 layers; particle size ~ 2 µm; thickness ~3–4 nm)	Intratracheal instillation: 1.25, 2.5 and 5 mg kg^-1^	90 days	ICR mice	Blood and BAL analysis: concentrations of pro-inflammatory cytokines (IL-1β, TNF-α, IL-6, IL-2, Th1-type cytokines, Th2-type cytokines) and chemokines (MIP)-1α, MCP-1, and GM-CSF in BAL fluids and immunoglobulins (Ig, IgE, IgG, and IgM) in serum. Expression of genes encoding actin family cytoskeletal proteins, calcium-binding proteins, and natriuretic-related genes. Histopathological analysis of lung.	BAL: increased number of lymphocytes, GNP-engulfed macrophages and apoptotic cells; general increase in cytokine and chemokine secretion; blood: increased number of macrophages and neutrophils, and elevated production of IgG, IgM and IgA. Gene expression: elevated expression of gens related to actin family cytoskeletal proteins and calcium-binding proteins; and alteration of natriuretic-related genes expression. Histopathological analysis: presence of GNP-engulfed macrophages without pathological lesion	(119)
Single- and multi-layered GO (SLGO and MLGO) in the presence or absence of Pluronic F-127	10, 20, 40, 80 and 100 μg ml^−1^	6 h	THP-1 cells	Cell viability, membrane integrity, cell morphology levels of cytokine and ROS production, phagocytosis, and cytometric apoptosis.	SLGO induced ROS and IL-1β production, necrosis, and apoptosis to a lesser extent than MLGO. However, SLGO induced higher membrane damage and decrease in cell viability.	(120)
	Iv: 10 mg kg^-1^	24 h (acute toxicity) or 10 days (chronic toxicity)	Mice	Histological analysis of lung and kidney: immunohistochemistry (IHC) for MCP-1 and TGF-β.	Both SLGO and MLGO induced acute and chronic damage to the lung and kidney in the presence or absence of Pluronic F-127.	
GO-PEG with mean thickness of 1.1 nm and lateral dimension ranged from 20 to 80 nm	It: 25 mg/kg	28 days	Balb/c mice: Age: 6 - 8 weeks; Weight: 18–22g	Blood circulation test; Hematologic and Biochemical marker analysis; Histopathological evaluation: trace element biodistribution observation in heart, liver spleen, lung, kidney and lymph.	Blood exposure to GO under the maximum safe starting dose caused accidental death in 1/5 *Macaca fascicularis* and 7/221 mice, while remains general amenable in others. Elevated levels of immunoglobulin E and severe lung injury were found in dead animals, suggesting the GO-induced acute anaphylactic reactions.	(121)
	4 mg/kg	90 days	*Macaca fascicularis*:Age; 4–5 years; weight: 4–5 kg			
Graphene oxide – silver nanoparticles hybrid material (GOAg)	5, 10, and 25 mg mL^−1^	24 h	J774 and primary murine macrophages	Cell viability, apoptosis/necrosis, mitochondrial depolarization, lipid peroxidation, cytokines release (IL-1β, TNF-α and IL-10), ratio between CD80 and CD206 macrophage populations and NO production.	GOAg induced a dose-dependent mitochondrial depolarization, apoptosis, and lipid peroxidation to J774 macrophages. However, no effects were observed on cytokines release, macrophages polarization toward M1 and NO production.	(122)
Bimetallic oxide FeWOx -PEG nanosheet (FeWOx-PEG)	0-200 μg ml^−1^	24 h	4T1 and CT26 cells	Cell viability, internalization, ROS generation.	No significant toxicity was observed, however FeWOx-PEG could internalize *via* cell endocytosis and efficiently active OH generation and GSH depletion.	(123)
	Toxicity: 10 mg kg^−1^ Biodistribution: 120 mg kg^−1^		BALB/c mice	Body weight, histological analysis, blood chemistry, cytokines secretion (IL-6, IL-12 and TNFα) and biodistribution.	No significant differences in blood chemistry were observed for FeWOx-PEG treated mice. Also, H&E staining and histology analysis showed no obvious tissue damages and adverse effects and no significative body weight changes. However, FeWOx-PEG induce strong immune responses, showed by the increase levels of IL-6, IL-12 and TNFα. Biodistribution analyses showed that the material could accumulate in liver and spleen, however, it was observed a decrease concentration after 7 and 14 days indicating the biodegradable and clearable behavior of FeWO_X_ -PEG nanosheets.	
FePSe_3_@APP@CCM	0-160 μg ml^−1^	Viability: 6 h Cytokine secretion: 48 h	PBMC, CT26 and RAW-264.7 cells	Viability and cytokines secretion (IL-10, IL-12 and IFN- γ)	No obvious cytotoxicity was caused by the nanomaterial However, taken together, upon NIR laser irradiation, FePSe_3_@APP@CCM matured and activated immature DCs, enhanced the secretion of IFN-γ and IL-12, and decreased the expression and the consequent inhibitory effect of IL-10 on T cells, resulting in the enhanced immunity of T cells for killing CT26 cancer cells in the coculture system.	(124)
	10 mg kg^−1^	25 days	C57BL/6J mice	Body weight, blood biochemical parameters (ALT, AST, BUN, CRE, LDH and PLT), histological analysis and cytokines secretion.	No obvious abnormality, inflammation and exudation or other pathological lesions were observed. Also, it was observed the increased expression of DC-secreted cytokines, including IFN-γ and IL-12, while the level of IL-10 was found to be decreased.	
Ferrimagnetic vortex-domain iron oxide nanoring and graphene oxide (FVIOs-GO) hybrid nanoparticle	50 or 75 μg ml^−1^ Fe	8 and 24 h	4T1 breast cancer cell and RAW264.7	Cell viability, uptake, apoptosis/necrosis, ROS generation, macrophages polarization.	Increased ROS generation and macrophage polarization to pro-inflammatory M1 phenotypes.	(125)
	Iv: 3 mg kg^−1^	24 days	Balb/c mice Subcutaneous 4T1 Breast Tumor Model	Measurement of tumor width and length for 24 days.	Control group exhibited a rapid increase in the tumor volume, while FVIOs-GO group had tumor growth inhibition by 97.1%.	
Borophene nanosheets (B NSs), graphene nanosheets (GR NSs) and phosphorene nanosheets (BP NSs)	Viability: 60, 80, and 100 μg ml^−1^ Membrane damage: 100 μg ml^−1^ Uptake: 200 μg ml^−1^	Viability: 24 h Uptake: 6 h	dTHP-1 and SC cells	Cell viability, membrane damage, cell uptake, intracellular localization, inflammatory cytokines secretion (IL-1β, IL-6, IL-8, IFN- γ and TNFα).	Corona coated 2D monoelemental nanosheets decreases cytotoxicity and cell membrane damage. For B NSs it was observed an increase in cellular uptake when the material was coronated, therefore corona may promote phagocytosis. Protein corona also stimulates the secretion of inflammatory cytokines. GR NSs and B NSs had immunoregulation behaviors only in the presence of plasma corona, while BP NSs had stronger immunoregulation behavior regardless of the absence and presence of corona.	(126)
Aggregated MoS_2_ and 2D MoS_2 (_exfoliated by lithiation or dispersed by Pluronic F87)	6.25–50 μg ml^−1^	24 h	THP-1 and BEAS-2B cells	Measurement of IL-8, TNF-α, and IL-1β levels	Aggregated MoS_2_ induced significant increases in IL-8, TNF-α, and IL-1β production, while there were significantly less effects of 2D MoS_2_ on cytokine and chemokine production.	(127)
	2 mg kg^−1^	40 h and 21 days	C57Bl/6 mice	BALF and lung tissue were collected for measurement of LIX, MCP-1, IL-6, TGF-β1, and PDGF-AA levels and performance of Hematoxylin and Eosin (H&E) or Masson’s trichrome staining.	Aggregated MoS_2_ induced robust increasing in LIX, MCP-1 and IL-6 responses along with neutrophilic exudation into the BALF; while 2D MoS_2_ did not trigger cytokine or chemokine production in the lung. Histopathological changes were observed with aggregated MoS_2_ inducing focal areas of inflammation around small airways, while 2D MoS_2_ had little or no effect.	
Exfoliated pristine and covalently functionalized MoS_2_	1, 10, 25, 50, 75, and 100 μg ml^−1^	24 h	Raw-264.7 and human monocyte-derived macrophages	Cell viability, CD86 expression and secretion of TNFα and IL6.	Cell viability was reduced only at high concentration; no variation of CD86 levels in both RAW 264.7 cells and human monocyte-derived macrophages was registered; no increase in cytokine secretion was observed for both cell lines.	(128)
Pristine MoS_2_ and PEGylated MoS_2_	10 μg ml^−1^	24 h	Primary mouse macrophages	Cytokine secretion (IL-6, IL-10, MCP-1, IFN-γ, TNF-α and IL-12).	Both materials significantly increased the secretion of cytokines such as IL-6, IL-12, TNF-α, IFN-γ and MCP-1. Interestingly, MoS_2_-PEG was found to elicit stronger cytokine secretion than the pristine MoS_2_, particularly involving IL-6, TNF-α, IFN-γ, and MCP-1.	(129)
MoS_2_ alone, MoS_2_–PEG or MoS_2_–PEG–CpG	0, 5, 10, 20, 30, 40 and 50 μg mL^-1^	48 h	RAW-264.7 cells and 4T1 cells	Cell viability, Cytokine release (TNF-α and IL-6),	MoS_2_ alone, MoS_2_–PEG or CpG alone had no effect on cytokine release while the MoS_2_–PEG–CpG significantly elevate the cytokine level. MoS_2_–PEG–CpG could elevate the expression of CD86 & CD80 and the percentage of matured DCs (CD80+ CD86+ DCs) was remarkably raised to 79.8% when combined with NIR irradiation.	(130)
Protein coated with different proteins (HSA, Tf, Fg and IgG) MoS_2_ NSs	500 μg ml^−1^	12 and 24 h	THP-1 cells	Cellular viability, cellular uptake and cytokine release.	Protein coated MoS_2_ NSs increase viability and decrease cytoplasmic membrane damage comparing with MoS_2_ NSs. Also, the presence of a protein corona decreased the secretion of cytokines. Among the four NSs the IgG coated MoS_2_ NSs enhanced uptake and cause more inflammatory cytokines.	(31)
MoS_2_ nanosheets (100 and 500 nm)	0 – 128 μg ml^−1^	48 h	DC cells	Cell viability, apoptosis, ROS generation, expression of CD40, CD80, CD86 and CCR7, secretion of proinflammatory cytokines (IL-12p70, IL6, IL-1β and TNF-α, DC homing ability.	Overall, there were no significant differences in cytotoxicity assays, however high doses could promote DC maturation as observed by the expression of CD40, CD80 and CD86 and enhanced secretion of IL-6 and TNF- α. Also, MSNs upregulate ROS generation in DCs, further promoting cytoskeletal rearrangement and promoting the local lymphoid homing ability of DCs.	(131)
Black phosphorus nanosheet (BPNSs) and black phosphorus quantum dot (BPQDs) (~300 nm)	100, 50, 25, 12.5 μg ml^−1^	48 h	H1299, L0-2, 293T, THP-1 cell line and SC human macrophages	Cell viability, cellular uptake (1, 3, or 6 h), intracellular localization, ROS generation, cytokines release (IL-1 β, IL-6, IL-8, IL-9, IL-10, IFN- γ), NO and TNF- α generation.	A reduction of cytotoxicity was observed when BPNSs and BPQDs were coated with protein corona reduced. However, the corona facilitated the BP internalization and induced an increase in inflammatory cytokines and in ROS generation. Also, an induction of NO and TNF- α production were provoked by BP and corona coated BP.	(132)
Black phosphorus nanosheet (128 nm)	15 μg ml^−1^	24 h	4T1, F10, CT26 and Raw-264.7 cell lines	Cell morphology, cell expression differences, expression of the surface marker CD80 using flow cytometry, proteomic analysis, western blot analysis and immunofluorescence to analyze, expression of IL-10 (M2-related marker) and TNF- α (M1-related marker).	Corona coated black phosphorus nanosheet increase the expression of calcium signaling pathways and interact with STIM2 protein facilitating Ca^2+^ influx promoting macrophage polarization.	(133)
Few-layer two-dimensional black phosphorous (2D BP)	10 to 500 μg.ml^−1^	24 h (acute toxicity) or 21 days (chronic toxicity)	SAOS-2, HOb, L929 and hMSC cell lines	Cell viability and proliferation, ROS production, immunofluorescence to analyze cell morphology, inflammatory marker expression tested by LPS to analyzed cytokine generation (IL-10 and IL-6).	Black phosphorus did not show cytotoxicity on human mesenchymal stem cells and inhibits the metabolic activity of SAOS-2 cell line while inducing both proliferation and osteogenic differentiation in HOb cell and mesenchymal stem cells. Also, the presence of BP inhibits the ALP (an early marker of osteogenesis) expression in SAOS-2 cells and induces antiproliferative and apoptotic effects by increasing the production of ROS on SAOS-2 cells. Besides, increase the inflammatory cytokine generation but inhibits proinflammatory mediators for the co-culture of SAOS-2 and HOb.	(134)
Black Phosphorus nanoflakes functionalized with TGF-β inhibitor and neutrophil membrane (NG/BP-PEI-LY)	20 μg ml^−1^	24 h (*in vitro*)	4T1 and HUVEC cell line	Cell viability, ROS production, apoptosis, cytokine generation (IL-6 and TNF-α)	NG/BP-PEI-LY induced acute inflammatory responses, cause a decrease in viability, and increase apoptosis and ROS production when laser irradiated.	(135)
		72 h (*in vivo*)	BALB/c mice	Mice NIR fluorescent imaging, immunofluorescent staining of CD31 (red) and ICAM-1 (green).	Besides, when laser irradiated increased the ICAM-1 expression, enhancing intracellular delivery by adhesion molecule mediated targeting.	
Black Phosphorus nanosheet (BPNS) and Black Phosphorus nanocomposite (BPCP) modified with PEG and OD CpG or CpG-Cy5.5	Up to 100 μg ml^−1^	24 h	4T1, RAW-264.7 and Hep62	Cell viability, necroptosis, protein expression, cytokine generation (IL-6 and TNF-α) and hemocytolysis.	No obvious cytotoxicity was observed, also no significant hemolysis. For BPTT treatments it was observed that necroptosis play an important role, mediating death process in cancer cells. These results were confirmed by the expression of necroptosis-related proteins, where it was observed a significantly expression of RIP1 and RIP3. Caspase-8 and Caspase-3 levels were not significantly changed.	(136)
	2 mg/kg	Up to 16 days	BALB/c mice	Biodistribution, expression of immune factors (FOXP3, IL-2, TNF- α and INF- γ), histological analysis, hematological toxicity.	No body weight loss and no systemic toxicity were observed. Also, no tissue damage and blood physiological indicators were within normal range. After BPTT treatments the immune responses were activated as observed by detection of T lymphocytes and various immune cytokines.	
DSPE-PEG coated Tao nanosheet (92.5 nm)	1 mg ml^−1^	30 days	C57 mice	Body weight, biodistribution, immunogenicity, hematological toxicity, liver and spleen histopathology, oxidative stress response.	DSPE-PEG coated TiO_2_ nanosheet cause a decrease in body weight after 14 to 30 days of the injection, also, it was observed a that the particles were accumulated in liver and cause liver toxicity by inducing oxidative stress. Besides, an obvious decrease in HTC and significant increase in MCH and MCHC indicate that the particles may induce blood system damage.	(137)
Two-Dimensional Core – Shell MXene@Gold Nanocomposites	*In vitro*: 3.1 to 100 μg ml^−1^	24 h	4T1 cell line	Cell viability, immunohistochemistry and immunofluorescence staining.	Overall, the particle did not show apparent cytotoxicity, and no toxic side effect was observed in mice after 30 days of injection.	(138)
	*In vivo*: 20 mg kg^-1^	30 days	Balb/c mice	Body weight and biodistribution.	No height loss and no notable abnormality on major organs were observed.	
2D titanium nanosheets (TiNS) and polyethylene glycol coated titanium nanosheets (TiNS-PEG)	*In vitro*: 10-100 ppm	4 h	A1 cell line, J774A.1 cell line and SMMC-7721.	Cell viability.	TiNS and TiNS-PEG did not significantly affect cell viability.	(139)
	*In vivo*: 5 mg kg^-1^	19 days	Balb/c mice	Histopathology, body weight, biodistribution and hematological toxicity.	Any significant differences on mice body weight, no histological abnormalities, and no impact on hematological parameters, indicating no inflammation and other negative impact on blood and organs was observed.	
PEGylated molybdenum dichalcogenides (MoS_2_-PEG), tungsten dichalcogenides (WS_2_-PEG) and titanium dichalcogenides (TiS_2_-PEG) nanosheets	*In vitro*: 25 – 200 μg ml^−1^	24 h	RAW-264.7, 4T1 and 293T.	Cell viability and ROS generation.	No significant *in vitro* cytotoxicity was observed for all the three types of PEG functionalized TMDCs.	(140)
	*In vivo*: 10 mg kg^-1^	up to 60 days post injection	Balb/c mice.	biodistribution, hematological toxicity, biochemical parameters (ALP, ALT, AST and BUN) and histopathology.	The materials show dominate accumulation in reticuloendothelial systems (RES) such as liver and spleen after intravenous injection. Also, no significant results were observed for the analyzed biochemical and hematological parameters and no obvious sign of abnormality, such as inflammation, was noticed in all examined major organs.	
Two-dimensional polyethylene glycol modified TiS_2_ nanosheets (TiS_2_-PEG)	*In vitro*: 0.0015 - 0.1 mg ml^−1^	24 h	4T1 cell and	Cell viability	No significant cytotoxicity of TiS_2_-PEG was observed.	(141)
	*In vivo*: 20 mg kg^-1^	60 days	Balb/c mice	*In vivo* toxicity, histopathology.	No histological abnormalities and no obvious toxicity to Balb/c mice was observed.	
BSA coated 2D silicene nanosheets (SNSs-BSA)	*In vitro*: 12.5 - 200 μg ml^−1^	24 h	4T1 and U87 cell lines	Cell viability	SNSs-BSA exhibit insignificant effect on cell viability of either 4T1 or U87 cancer cells.	(142)
	*In vivo*: 20 mg kg^-1^	4 weeks	Kunming mice and Balb/c mice	Body weight, histopathology, hematological toxicity, biochemical parameters (ALT, AST, ALP, urea, CREA, and UA).	In a four-week duration, the mice present no significant abnormality, body weight differences, and no significant behavioral alterations. The histological observations of major organs showed no significant acute pathological toxicity. Furthermore, hematological parameters showed no obvious sign of abnormalities indicating that the SNSs-BSA induce negligible renal and hepatic toxicity in mice model.	
Poly(vinylpyrrolidone)-encapsulated Bi_2_Se_3_ nanosheets (diameter 31.4 nm and thickness 1.7 nm)	*In vitro*: 5 - 200 ppm	48 h	MCF7 cell line	Cell viability	It was not observed any cytotoxicity effects caused by Bi_2_Se_3_ nanosheets.	(143)
	*In vivo*: 27 – 1168 mg kg^-1^	14 days	Balb/c mice	*in vivo* toxicity and biodistribution.	At the dose of 750 or less no mice mortality nor any reaction was observed. The nanomaterial mainly accumulated in liver, spleen and kidney, however, the concentration decreases with time.	
Pd nanosheets (diameter ranging from 5 to 80 nm)	*In vitro*: up to 100 μg ml^−1^	24 h	NIH-3T3, 4T1, Raw-264.7, QSG-7701 and QGY-7703 cell lines	Cell viability, mitochondrial membrane depolarization and ROS generation.	Pd nanosheets have no effect on cell viability, apoptosis, ROS generation, or mitochondrial depolarization.	(144)
	*In vivo*: 10 mg kg^−1^	30 days	Balb/c mice	Biodistribution, blood chemistry and hematology analysis and histopathology.	The *in vivo* results show that the particle is primarily trapped by reticuloendothelial system (RES). Also, no significant hepatotoxicity was induced by Pd nanosheets of different sizes. The activity of ALP, ALT, AST and BUN observed was within normal range and no apparent histopathological abnormalities or lesions were observed in any major organ.	
PEGylated ultrathin boron nanosheets (B-PEG NSs)	25 to 500 µg mL^−1^	48 h	HeLa, PC3, MCF7, and A549	Cell viability, ROS generation.	No significant cytotoxicity was observed for B-PEG NSs. However, when exposed to an 808 nm NIR laser (1 Wcm^−2^) for 5 min it was notices a strong concentration-dependent cytotoxicity. Also, when the B-PEG NSs were combined with DOX and NIR laser irradiation, over 95% of the cells died at a DOX concentration of 100 µg mL^−1^.	(89)
	5.3 mg kg^-1^	24 h	Mice	Body weight, histopathology, hematological toxicity (HGB, WBC, RBC, MCV, MCHC, PLT, MCH, HCT, Cr, NEU, LYM, MPV), biochemical parameters (ALP, AST, BUN and ALT) and cytokine generation (TNF-α, IL-6, IFN-γ, and IL-12+P40)	No obvious side effects were noted, also the levels of TNF-α, IL-6, IFN-γ, and IL-12+P40 were similar to those in the PBS control group indicating that B-PEG NSs did not induce obvious cytokine response. Compared with the control group, there is no statistically significant difference of the NSs-treated groups with PBS-treated groups in all the parameters, no obvious induction on cytokine response, no change in biochemical parameter and no hematological toxicity, therefore, B-PEG NSs do not cause obvious infection and inflammation in the treated mice. Moreover, no noticeable signal of inflammation or tissue damage was observed in major organs.	

^1^Ip, intraperitoneal; ^2^Lung, oropharyngeal aspiration; ^3^It, intratail.

GO-PEG, poly-(ethylene glycol)-functionalized GO; PG-FMN, flavin mononucleotide-stabilized pristine graphene; GO-NH2, aminated GO; GO-PAM, poly(acrylamide)-functionalized GO; GO-PAA, poly(acrylic acid)-functionalized GO; PEG, polyethylene glycol; DSPE-PEG, N-(carbonyl-methoxypolyethyleneglycol 5000)-1,2-distearoyl-sn-glycero-3-phosphoethanolamine; HSA, human serum albumin; Tf, transferrin; Fg, fibrinogen; IgG, immunoglobulin G; NSs, Nanosheets; ALP, aspartate aminotransferase; ALT, alanine aminotransferase; LDH, lactate dehydrogenase; BUN, blood urea nitrogen; CRE, creatinine; lactate dehydrogenase; PLT, platelet; NO, nitric oxide; IHC, immunohistochemistry; Nuclear NMR, magnetic resonance spectroscopy; TCA, tricarboxylic acid cycle; PVP, polyvinyl chloride; LPS, lipopolysaccharide; Rho/ROCK, Rho-associated protein kinase; RBC, red blood cells; WBC, white blood cells; MMP, mitochondrial membrane potential; MAPKs, mitogen−activated protein kinase; ERK, extracellular signal-regulated kinase; JNK, c-Jun N-terminal kinase; GSH, glutathione; BALF, bronchoalveolar lavage fluid; LYM, lymphocytes; MPV, mean platelets volume; HTC, hematocrit count; HGB, hemoglobin; MVC, mean volume cell; MCH, mean cell hemoglobin; MCHC, MCH concentration; NEU, neutrophil count; DOX, doxorubicin; NIR, near infrared light; UA, uric acid; CpG, cytosine–phosphate–guanine; BPTT, black phosphorus based photothermal therapy; TMDC, transition metal dichalcogenides.

Studies have demonstrated that 2D materials can induce immunological system activation with a consequent induction of an inflammatory response (145). This immunological system activation showed itself to be dependent of the 2D materials’ physicochemical properties, such as size (106–109, 144), surface chemistry (114, 115, 123), number of layers, shape (118, 119), and functionalization (109, 112, 114, 128, 135, 139). For example, Yue et al. (106) demonstrated that larger graphene oxide (GO) (2 µm) has induced a higher immunological activation than smaller GO (350 nm) both *in vitro* (peritoneal macrophages) and *in vivo* (C57BL/6 mice). Similarly, Ma et al. (107) showed a lateral-size-dependent pro-inflammatory effect of GO under *in vitro* and *in vivo* conditions, wherein the largest GO (L-GO; 750–1300 nm) elicit higher inflammatory response than smallest GO (S-GO; 50–350 nm). Moreover, the mechanism of inflammation has also differed according to the lateral size, with L-GO being more prone to plasma membrane adsorption and the toll-like receptors (TLRs) and nuclear factor-κB (NF-κB) pathways activation, whereas S-GO was mostly taken up by macrophages. In another study that investigated the effects of small GO (S-GO < 1 µm) and large GO (L-GO, 1–10 µm) on human peripheral immune cells, it was found that the S-GO has a more significant impact on the upregulation of critical genes implicated in immune responses and the release of cytokines IL1β and TNFα compared to L-GO (108). However, it is important to clarify here that the S-GO in this study presented similar lateral size of the L-GO in the previous studies cited, which means that all these studies are in agreement, and we may erroneously interpret them because attention to the lateral size was not devoted. Indeed, a nomenclature harmonization of GBMs is urgently needed to allow a clear understanding on the impacts of GBM physicochemical properties on their biocompatibility.

Besides to assess the effect of lateral size, Duarte and coworkers (109) investigated the impacts of two different surfaces functionalization: pegylated graphene oxide (GO-PEG, 200–500 nm) and flavin mononucleotide-stabilized pristine graphene with two different sizes (200–400 nm and 100–200 nm). Their results showed that the cellular uptake of GBMs was mainly influenced by their lateral size, with smaller particles showing greater internalization, while the inflammatory response depended also on the type of functionalization, with GO-PEG showing the lower pro-inflammatory potential. This study corroborates in number previous ones that also showed an increased biocompatibility of GO due to the pegylation (GO-PEG) (110, 111). Similarly, Xie et al. (139) studied PEG coated 2D titanium nanosheets (TiNS-PEG) and reported no indication of inflammation and other negative impacts. Moreover, the material was promising for photothermal tumor therapy and presented a high contrast for *in vivo* imaging. However, Gu et al. (129) found that MoS_2_ and PEGylated MoS_2_ induced a robust macrophage immune response, with PEG-MoS_2_ eliciting stronger cytokine secretion than the pristine MoS_2_. By performing molecular dynamics simulations, they demonstrated that small MoS_2_ nanoflakes can penetrate the macrophage membrane, and that the PEG chain on PEG-MoS_2_ lead to a prolonged passage throughout the membrane. Such a result might explain why PEG-MoS_2_ triggers sustained more stimulation of macrophages than pristine MoS_2_.

Other types of functionalization have also been studied in respect to their biocompatibility to immune cells. For instance, Zhi et al. (112) reported that the polyvinylpyrrolidone (PVP) coating of GO has exhibited lower immunogenicity when compared with pristine GO in relation to the inducing differentiation and maturation of dendritic cells (DCs), provoking a delaying in apoptotic process of T lymphocytes and the anti-phagocytosis ability against macrophages.

Surface chemistry has also been shown to influence on the immunotoxicity of 2D materials. Gurunathan et al. (114) reported that both GO and reduced GO (rGO) induced a dose-dependent loss of cell viability and proliferation, cell membrane damage, a loss of mitochondrial membrane potential, a decreased level of ATP, a redox imbalance, and an increased secretion of various cytokines and chemokines (IL1-β, TNF-α, GM-CSF, IL-6, IL-8, and MCP-1) by THP-1 cells. However, to all these toxic effects the rGO presented a significantly worse response compared to GO. In a previous study, Yan et al. (115) showed that different oxidation degrees resulted in the toxicity of monocytes *via* different signaling pathways, with GO nanoplatelets (GONPs) inducing the expression of antioxidative enzymes and inflammatory factors, whereas the reduced GO nanoplatelets (rGONPs) activated the NF-кB pathway. The contradictory results between these two studies, in relation to cytokine and chemokine expression, may be due to differences in the GBMs studied (i.e. GO sheets *versus* GO nanoplatelets), and they raise the need for further investigation concerning the effects of the oxidative degree of GBMs on immune cells.

In order to investigate the pristine graphene effects *in vitro* (THP-1 cell line) and *in vivo* (C57BL/6 strain mice), Schinwald et al. (118) have assessed the impacts of the shape of graphene nanoplatelets (GNPs) on their inflammatory potential. This large few-layer graphene presented as inflammogenic both *in vitro* and *in vivo*, which was attributed to its large size that led to frustrated phagocytosis. The authors highlighted that the potential hazard of GNPs could be minimized by producing GNPs small enough to be phagocytosed by macrophages. Moreover, the number of GO layers has been shown to affect its immunotoxicity, in which single-layer GO (SLGO) caused a more pronounced decrease in cell viability due to membrane damage of THP-1 cells, while multi-layer GO (MLGO) induced higher reactive oxygen species (ROS) and IL-1β production, leading to necrosis and apoptosis (120). In addition, the histological animal analysis revealed that SLGO and MLGO induced acute and chronic damage to the lungs and kidneys in the presence or absence of Pluronic F-127 (120).

Another important parameter, when approaching nanomaterial biosafety, is colloidal stability. Aggregation can influence the immunological response as observed by Wang et al. (127), when compared the toxicological profile of 2D MoS_2_ versus aggregated MoS_2_ in lung cells and mice. In their *in vitro* evaluation, in THP-1 and BEAS-2B cells, they found that aggregated MoS_2_ induces strong proinflammatory and profibrogenic responses, while 2D MoS_2_ have little or no effect. In agreement with *in vitro* results, an acute toxicity study *in vivo* showed that aggregated MoS_2_ induced an acute lung inflammation, while 2D MoS_2_ had no or a slight effect.

To increase the stability of 2D materials, studies have shown that proteins can be used as a dispersant agent. Lin et al. (142) studied silicene nanosheets modified with a bovine albumin serum protein corona (SNSs-BSA) and observed a significant increase in the colloidal stability in several physiological media (0.9% saline, phosphate buffered saline and Dulbecco’s modified Eagle medium). Furthermore, SNSs-BSA did not cause significant toxicity *in vitro* neither significant acute toxicity *in vivo*. Only meaningless hematological changes were observed during the treatment duration, and no significant inflammation or infection were caused by the SNSs-BSA.

It is imperative that in a physiological environment, the nanomaterials will interact with biomolecules, forming a complex biomolecular corona. Those biomolecules (e.g., proteins, lipids, carbohydrates) can change the identity of the nanomaterials and influence their interaction with biological systems, causing an increase or decrease in internalization, toxicity, and biocompatibility as well as in colloidal stability over time. Thus, the biotransformation of nanomaterials in a physiological environment is an important parameter to be studied (146). The most common and highly studied component of biomolecular corona is the protein corona. In this sense, Mo et al. (132) studied the effect of the human plasma protein corona on the cytotoxicity of BP nanosheets and BP quantum dots (BPQDs) observing a reduction in cell viability for both nanomaterials when coated with proteins. However, protein corona facilitated BP nanosheet internalization and induced an increase in inflammatory cytokines (IL-1β, IL-6, IL-8 and IFN-γ) and in ROS generation. Besides, it was observed that protein corona coated BP caused an induction on the nitric oxide (NO) and tumour necrosis factor. Further, Mo et al. (133) studied the effect of the human plasma protein corona in BP toxicity, and observed an increased macrophage polarization due to the adsorption of opsonins present in the plasma, increasing the uptake of BP and the interaction with stromal interaction molecule 2 (STIM2) protein facilitating Ca^2+^ influx.

Similarly, Han et al. (126) studied the effect of plasma corona-coated 2D monoelemental nanosheets and observed that the protein corona decreases cytotoxicity and cell membrane damage for borophene, phosphorene, and graphene nanosheets. The corona coating induced the secretion of inflammatory cytokines (IL-1β, IL-6, IL-8, and IFN-γ) for all three materials. Also, for BNNs, it was observed an increase in cellular uptake when the material was coronated, and therefore, the corona may promote phagocytosis. Baimanov et al. (31) also investigated the effect of four different blood protein coronas (human serum albumin (HSA), transferrin (Tf), fibrinogen (Fg), and immunoglobulin G (IgG) corona) on cell viability, uptake, and pro-inflammatory effects of MoS_2_ nanosheets (NSs) in the macrophages cell line. Their results demonstrate that blood proteins contribute to uptake and inflammatory effects, as protein coated MoS_2_ NSs increase cell viability and decrease cytoplasmic membrane damage when compared to non-coated MoS_2_ NSs. Besides, it was observed that the type of protein influences cytokine secretion, as IgG-coated MoS_2_ NSs causes more inflammatory cytokine secretion (TNF-α, IL-6 and IL-1β). The highest proportion of β-sheets on IgG led to fewer secondary structure changes on MoS_2_ NSs, facilitating uptake and producing a stronger pro-inflammatory response in macrophages due to the recognition of an MoS_2_ NSs−IgG complex by Fc gamma receptors and the subsequent activation of the NF-κB pathways. Another interesting finding is that in a serum-containing medium, cellular uptake of MoS_2_ NSs−protein complexes was higher than that in a serum-free medium. Also, the MoS_2_ NSs−Fg, and MoS_2_ NSs–serum complexes had similar results in serum-free conditions and different results in a serum-containing medium, suggesting the formation of the protein corona layer above the previously formed MoS_2_ NSs−protein complexes. Those studies can help to elucidate the mechanisms in which protein corona can affects the toxicity of 2D materials.

One important ability of the immune system is the innate immune memory, where cells from the innate immune system react to secondary stimulus, which mostly includes an increased or decreased production of inflammation-related factors (147). With regard to 2D materials studies, there is yet a little research on this topic. Liu et al. (148) functionalize GO with lentinan (LNT) and observed that GO-LNT was able to promote macrophage activation by NF-κB and TLR signaling pathway, as well as enhance antigen protein processing after initial contact with macrophage. Moreover, the efficiency of this material was investigated, as a vaccine adjuvant for ovalbumin (OVA), in this sense GO-LNT induced robust long-term OVA-specific antibody responses due to the prolonged release of OVA. Besides this, GO-LNT was able to sustain a long-term immune response because it facilitated the uptake and slowed the release rate of antigen in macrophage. Further, Lebre et al. (149), demonstrated that pristine graphene can promote the innate immune training, enhancing the secretion of IL-6 and TNF-α and a decrease in IL-10 after toll-like receptor ligand stimulation 5 days after graphene exposure, indicating that pristine graphene can activate the immune innate memory.

Immune cells, such as macrophages and neutrophils, are one of the first line of defense of the immune system; they are capable of engulf the foreign material (or pathogen), degrading it and producing cytokines to enhanced the immune response (150). The uptake of 2D materials by immune system cells have been reported in various studies (31, 109, 115, 126, 132); however, there are few studies that address the degradation of those materials after internalization. Mukherjee et al. (151) studied the degradation of large and small GO by neutrophils and observed that not only both GO be degraded by neutrophils but also that the product of the degradation was non-toxic to human cells. Similarly, Moore et al. (152) studied the degradation of few-layer MoS_2_ in human macrophage-like cells and observed that internalization occurred following 4 h of exposure and after 24 h the *in vitro* degradation of the material was confirmed, which occurred within lipidic vesicles and associated with enzymatic regions containing lysozyme.

As presented above, 2D nanomaterials may have an inflammogenic potential and immunotoxicity, which may impair their successful clinical translation; however, the immunological system activation can also be useful for theragnostic purposes. This application uses the immune responses to protect the body and eliminate cancer cells. The advantage of immunotherapy is that it engages the immune system to kill tumor cells without damaging healthy cells, additionally, it may induce immunological memory, causing long-lasting protection (153).

### Nanoinformatics Approaches Toward Immunosafety-by-Design

In materials science, theory, computational modeling and informatics have a substantial role in accelerating and discovering new materials with interesting properties and applications (154–156). Due to the growing interest in 2D nanomaterials, computational approaches are extensively used in the discovery, development and application of these materials by detailed study of their structure/property relationships (156–158).

The nano-bio interface phenomena are directly related to the physicochemical properties of nanomaterials. However, tracing general correlations and delineating predictive models of nanomaterials biological effects remains challenging. Some issues include the complexity of nano-bio interactions, nanomaterials structural heterogeneity, lack of standard methodologies, absence of systematic studies and low-quality nanomaterial characterization (159–161). In this context, computational methods have been incorporated into the nanotoxicology field to support the understanding of the nano-bio interface to enable the development of safe-by-design principles applied to nanomaterials (162, 163). Theoretical modeling (i.e., molecular dynamics, density functional theory) enables precise control of critical parameters of the nanomaterials surface to study their individual effects in nano-bio interactions, providing mechanistic knowledge (164–166). On the other hand, machine learning (ML) techniques are used to assess datasets of nanomaterials biological outcomes in order to find patterns and correlations between physicochemical properties and biological effects, often undetectable through other types of analysis (167–169).

Applications of data-driven strategies include data filling, grouping, and predictive modeling. Quantitative nanostructure–activity relationships (QNAR) consist of the main strategy to delineate prediction models based on correlations between nanomaterial structural characteristics to their properties and biological activities (170, 171). It is based on the assumption that nanomaterials in their properties present similar biological effects. Diverse algorithms can be used in QNAR models, including support vector machine (172), artificial neural network (173), and decision trees (174), among others, and depending of the level of algorithms interpretability may enable the outline of causal relationships.

The scarcity of quality data and comprehensive databases is the major bottleneck in the application of ML to predict nanomaterials immune reactions (175, 176). Data-driven strategies have been making important advances in modeling biological phenomena that have potential usage to evaluate nano-immune interactions, such as predicting biomolecular corona compositions (177–181), and nanomaterials and cell interactions (e.g., cell uptake, cytotoxicity, membrane integrity, oxidative stress) (182–185). Furthermore, the exploration of omics approaches (e.g., genomics, transcriptomics, and metabolomics) has promoting the development of ML models to process the complex data generated by these techniques and enables a better understanding of the molecular mechanisms of nanomaterials adverse effects in a systemic context, defining and predicting adverse outcome pathways (186–189). The omics’ potential of data generation is demonstrated by Kinaret et al. (190), who were able to connect immune responses to observed transcriptomic alterations in mouse airway exposed to 28 engineered nanomaterials. Together with cytological and histological analyses (imaging processing), they generated an extensive *in vivo* data set of nanomaterial adverse effects.

Allied with quality data infrastructure and processing, computational methods are sizeable to deal with complexity of nano-bio interface to assess and model the toxicity of nanomaterials in a variety of environments (163, 191–194). To support safe-by-design approaches, international efforts have been made to provide data integration and sharing, modeling tools, standard protocols, and ontologies, to ensure Findable, Accessible, Interoperate, and Reusable (FAIR) data (195, 196). For example, European projects, such as NanosolveIT and NanoCommons, and more recently CompSafeNano are initiatives facing on this direction (164, 165, 197, 198). In accordance with these initiatives, Gazzi et al. (199) recently presented the nanoimmunity-by-design concept developed inside G-IMMUNOMICS and CARBO-IMmap projects, which aim to bridge the knowledge gaps in the immune characterization of carbon-based materials, integrating data-driven methodologies which are extendable to other 2D materials.

## Conclusions And Future Perspectives

Two-dimensional materials are key elements for nanoscience and innovation in energy, health, and the environment. This can lead to a broad range of technological applications, especially nano-imaging, which has been growing exponentially in recent years. The wide number of 2D materials with different physicochemical properties make immunotoxicity and safety evaluation a challenge. There are therefore still gaps and controversial data in the literature. For example, within the same material category (i.e., graphene oxide) different properties were observed that might affect immunological and toxicological responses. It is imperative to evaluate the biological effects of biomolecular corona formation on 2D materials at nanobiointerfaces. Only by the identification of these material properties (intrinsic and extrinsic) and an integrated understanding on how they may influence its immunological response, we can manage immunotoxicity/biocompatibility and then benefit from their unique properties for many applications. Furthermore, it is very important to highlight the critical influence of endotoxin contamination prior immunological studies and toxicity testing. Special attention on this topic will avoid misinterpretation of immunosafety results involving 2D materials (148). In addition, it is important to advance in the understanding of the links between nanomaterials and the immune system across environmental species; this being a future challenge for immunosafety research associated with 2D materials (200). Nanoinformatics and computational modeling will have a decisive role on immunotoxicological studies with nanomaterials toward the practical implementation of immunosafety-by-design. However, it is very important to develop harmonized protocols, ontologies, and public databases to facilitate and promote a global research community for the collaboration and an exchange of knowledge in this field, focusing efforts on FAIR data principles.

## Author Contributions

All authors listed have made a substantial, direct and intellectual contribution to the work, and approved it for publication. GS and LFr: literature research, data curation, writing, and editing. RP, LFo, and MM: literature research and writing. DM, AF, and OA: funding acquisition, supervision, project administration, and writing. All authors contributed to the article and approved the submitted version.

## Funding

This work was funded by the Sao Paulo Research Foundation (FAPESP, grant no. 18/25103-0; 17/02317-2; 14/50906-9), the National Council for Scientific and Technological Development (CNPq, grant no. 315575/2020-4; 301358/2020-6), and the Coordination for the Improvement of Higher Education Personnel (CAPES, Finance code 001).

## Conflict of Interest

The authors declare that the research was conducted in the absence of any commercial or financial relationships that could be construed as a potential conflict of interest.

## Supplementary Material

The Supplementary Material for this article can be found onlineat: https://www.frontiersin.org/articles/10.3389/fimmu.2021.689519/full#supplementary-material
