# Supplementary material for: Recent Advances in Immunosafety and Nanoinformatics of Two-Dimensional Materials Applied to Nano-imaging
Source: Front Immunol. 2021 Jun 3;12:689519. doi: 10.3389/fimmu.2021.689519 (PMC8210669; doi:10.3389/fimmu.2021.689519)
Supplement: Supplementary file 1 [file DataSheet_1.docx]

Supplementary Material

# Supplementary Data

- 1. **Web of Science research methodology**

The literature review was performed on the Web of Science™ platform. The data was obtained on the Web of Science collection from the year 2000 to 2021, considering articles published. The queries used in these searches are detailed described below:

- - 1. **Search terms addressing 2D material applications**

Bone tissue engineering: TS = ((((two$dimens* OR 2D OR (two NEAR/2 dimens*)) NEAR/2 *material*) OR (2d *material*)) AND ((bone NEAR/2 tissue NEAR/2 engineering) OR (bone NEAR/2 regeneration) OR (bone NEAR/2 healing)))

Drug-delivery: TS = ((((two$dimens* OR 2D OR (two NEAR/2 dimens*)) NEAR/2 *material*) OR (2d material*)) AND ((drug?delivery) OR carrier OR *platform)))

Imaging: TS = ((((two$dimens* OR 2D OR (two NEAR/2 dimens*)) NEAR/2 *material*) OR (2d *material*)) AND ((nano$imag*) OR (nano* NEAR/2 imag*) OR (imag*)))

Sensing blood biomarkers: TS = ((((two$dimens* OR 2D OR (two NEAR/2 dimens*)) NEAR/2 *material*) OR (2d *material*)) AND (*sens* AND (blood NEAR/2 *marker*)))

Sensing water contaminants: TS = ((((two$dimens* OR 2D OR (two NEAR/2 dimens*)) NEAR/2 *material*) OR (2d *material*)) AND (*sensor* AND ((heavy NEAR/2 metal*) OR contaminant* OR pollutant*)))

Water desalination: TS = ((((two$dimens* OR 2D OR (two NEAR/2 dimens*)) NEAR/2 *material*) OR (2d *material*)) AND (*water NEAR/2 desalination))

Water remediation: TS = ((((two$dimens* OR 2D OR (two NEAR/2 dimens*)) NEAR/2 *material*) OR (2d *material*)) AND (*water NEAR/2 (remediation OR cleaning)))

Catalysis: TS = ((((two$dimens* OR 2D OR (two NEAR/2 dimens*)) NEAR/2 *material*) OR (2d *material*)) AND catalysis)

Energy storage: TS = ((((two$dimens* OR 2D OR (two NEAR/2 dimens*)) NEAR/2 *material*) OR (2d *material*)) AND (energy NEAR/2 (storage OR accumulation)))

- - 1. **Search terms addressing immutoxicity evaluation**

TS=((im$uno*safe* OR im$uno*toxic*) OR (im$une* NEAR/2 s$stem NEAR/2 activat*))

- - 1. **Search terms addressing 2D nanomaterials**

Nanomateriais 2D: TS = ((two$dimens* OR 2D OR (two NEAR/2 dimens*)) NEAR/2 nanomaterial*)

Graphene: TS = ((graphen* NOT (graphen* NEAR/2 oxid*))

Graphene oxide: TS = ((graphen* NEAR/2 oxid*)

Boron: TS = ((hexagonal$boron NEAR/2 nitrid*) OR (hexagonal* NEAR/2 boron NEAR/2 nitrid*) OR (2D NEAR/2 Boron))

Black phosphorus: TS = (black* NEAR/2 phosphorus)

Metals: TS = ((layer* NEAR/2 metal* NEAR/2 oxid*) OR (metal NEAR/2 dichalcogenid*) OR (metal NEAR/2 organic NEAR/2 sheet*) OR (coordination* NEAR/2 nanosheet*) OR (Manganes* NEAR/2 phthalocyanine NEAR/2 nanosheet*) OR (MnPc NEAR/2 nanosheet*) OR (2D NEAR/2 MnO2 NEAR/2 nanosheet*))

Lead: TS = (2D NEAR/2 Pd NEAR/2 nanosheet*)

Bismuth: TS = ((bismuth NEAR/2 tungstate NEAR/2 nanosheet*) OR (Bi2WO6 NEAR/2 2D) OR (bismuth NEAR/2 selenide NEAR/2 2D) OR (Bi2Se3 NEAR/2 nanosheet*) OR (Bi2O2Se NEAR/2 nanosheet*))

Ultrathin lanthanide oxyiodide: TS = (ultrathin NEAR/2 lanthanide NEAR/2 oxyiodide)

Titanium: TS = ((two$dimens* NEAR/2 titanium) OR (2D NEAR/2 Ti) OR (titanium NEAR/2 nanosheet*))

Rare-earth: TS = ((rare-earth NEAR/2 2D) OR (rare-earth NEAR/2 nanosheet*))

Silicene: TS = ((2D NEAR/2 silicene) OR (silicene NEAR/2 nanosheet*))

Antimonene: TS = ((2D NEAR/2 antimonene) OR (antimonene NEAR/2 nanosheet*))
