# Supplementary material for: Recent Advances in Immunosafety and Nanoinformatics of Two-Dimensional Materials Applied to Nano-imaging
Source: Front Immunol. 2021 Jun 3;12:689519. doi: 10.3389/fimmu.2021.689519 (PMC8210669; doi:10.3389/fimmu.2021.689519)
Supplement: Supplementary file 2 [file Table_1.docx]

**Table 1.** Relevant studies addressing the adverse immunological effects of 2D materials in *in vitro* and *in vivo* models from 2000 to 2021.

| **Nanomaterial** | **Dose** | **Exposure time** | ***in vivo*/*in vitro* models** | **Method or endpoints** | **Adverse immunological effects** | **Ref.** |
| --- | --- | --- | --- | --- | --- | --- |
| Graphene oxide (GO) (lateral size of 350 nm and 2 µm) | 2, 4 and 6 µg mL^-1^ | 24, 48, and 96 hours | peritoneal macrophage | Secretion of pro-inflammatory cytokines (IL-6, IL-10, IL-12, TNF-α, MCP-1, and IFN-ɤ) | Dose-dependent release of cytokines induced in a higher extent by 2 µm GO than 350 nm GO. | (108) |
|  | - | 21 days | C57BL/6 male mice | Histological micrographics | Mononuclear cells (i.e. macrophages and lymphocytes) infiltration and inflammation response induced by 2 µm GO, but not by 350 nm GO. |  |
| GO (smallest S-GO 50-350nm; intermediate I-GO 350-750 nm; largest L-GO 750-1300 nm) | Viability: 1-300 µg mL^-1^; Others:20 µg mL^-1^ | 12, 24 hours | J774.A1 and THP-1 macrophages | Live/dead assay, TNF-α, IL-6 and IL1β release; and macrophage polarization, NF-κB signaling activation. | All GO materials have induced a decrease in cell viability, and a production of cytokines. The L-GO significantly elicited higher response than S-GO. Higher macrophage polarization to the M1 phenotype by L-GO than S-GO. | (109) |
|  | *Ip^1^:* 5000 μg kg^-1^ bw; Lung^2^: 2500 μg kg^-1^ bw; It^3^: 5000 μg kg^-1^ bw | Ip: 72 hours; Lung: 72 hours; It: 24 hours | BALB/c male mice | Local and systemic inflammation: TNF-α, IL6 release, recruitment of immune cell. | Both S-GO and L-GO have induced an inflammatory response by cytokines production and leukocytes recruitment, been the L-GO response higher than the S-GO response in all endpoints. |  |
| GO  S-GO (<1µm)  L-GO (1-10 µM) | 25, 50, and 75 µg mL^-1^ | 24 hours | PBMCs, Jurkat and THP-1 cells | Annexin-V FITC (apoptosis), LIVE/DEAD FITC (late apoptosis and necrosis), and propidium iodide (necrosis), cell activation (expression of CD69 and CD25 markers), cytokine release, expression of 84 genes related to innate and adaptive immune responses | Only S-GO presented a decrease in cell viability at highest dose (75µg/mL). None of GO tested have induced the cell activation (expression of CD69 and CD25 markers). However, both GO induced cytokines release and upregulation of genes related to immune response, being that the S-GO response was significantly higher compared to L-GO response. | (110) |
| GO-PEG (200-500 nm) and PG-FMN (L) (200-400 nm) and PG-FMN (S) (100-200 nm) | 10 μg mL^-1^ | 24 hours | RAW-264.7 macrophages | Cellular uptake, nitric oxide production, NMR metabolic profiling, expression of cell surface markers CD80 and CD206. | PG-FMN (S) was internalized in a greater extent compared to GO-PEG and PG-FMN (L), which presented a similar uptake. GO-PEG did not induce NO production, whereas PG-FMN (S) and PG-FMN (L) caused significant NO increases of 21 % and 12 %, respectively. Only PG-FMN (S) caused increases in intracellular succinate and itaconate, similarly to LPS, while PG-FMN (L) did not alter the levels of TCA cycle intermediates and GO-PEG caused a decrease of succinate. Besides, GO-PEG decreased the TNF-α secretion compared to control cell, and do not affected the cell surface markers. | (111) |
| GO-PEG  (200-500 nm) | 40 and 80 μg ml^-1^ | 24 and 48 hours | Murine peritoneal macrophages | Cell surface markers of M1 (CD80 and iNOS) and M2 (CD206 and CD163) phenotypes. | PEG-GO did not induce the macrophage polarization towards the M1 pro-inflammatory phenotype, with a slight shift towards M2 reparative phenotype. | (122) |
| GO-1PEG  (~100 nm)  GO-6PEG  (~300 nm) | 2.3-75 µg ml^-1^ | 24 hours | RAW-264.7 macrophages and primary splenocytes (B-cells and T-cells) | Proinflammatory cytokine secretion (IL-1β, TNF-α and IL-6) and proliferation of immune cells. | Only GO-6PEG increased the secretion of TNF-α by RAW-264.7 macrophages without alteration of IL-6 and IL-1β levels. The treatment of primary splenocytes with GO-1PEG and GO-6PEG in the presence of concanavalin A, anti-CD3 antibody and LPS, produced significant dose-dependent decrease of cell proliferation and IL-6 levels. | (123) |
| GO and PVP coated-GO | 25, 50 and 100 µg mL^-1^ | 48 hours | Human DC, macrophages and T cells | Differentiation and maturation of DC cells, cytokine release, apoptosis of T cells, and phagocytosis | GO induced the differentiation and maturation of DC cells; a dose-dependent release of pro-inflammatory cytokines by DC cells; a dose-dependent apoptosis of T cells; and a susceptibility of phagocytosis by macrophages. The coating with PVP has reduced the cytokines secretion and the differentiation and maturation of DC cells; delayed the apoptotic process of T cells; and avoid the phagocytosis by macrophages. | (118) |
| GO  GO-NH_2_ ,  GO-PAM,  GO-PAA  GO-PEG | 1, 2, 4, 10, 20, 50, 100, or 200 μg mL^-1^ | 1, 6 and 24 hours | J774A.1 cell line | Viability, cellular adhesion, uptake, membrane permeability and fluidity, Ca^2+^ flux and transcriptome analysis. | GO caused the impairment of cell membrane integrity and functions including regulation of membrane- and cytoskeleton- associated genes, membrane permeability, fluidity, and ion channels. The -NH_2_ and -PAA showed similar toxicity to GO, but -PEG and -PAA significantly decreased the GO cytotoxicity. | (139) |
|  | It: 1mg kg^-1^ | 24 hours | Male BALB/c mice | Survival, body weight increase, complete blood count (numbers of RBC, WBC, PLT, neutrophils, lymphocyte), blood biochemistry, GO distribution, histological analysis of lung, liver and spleen. | GO induced platelet depletion, pro-inflammatory response and pathological changes of lung and liver in mice. The -NH_2_, -PAA and -PEG modifications greatly reduced the toxicity of GO in mice. The -PAM modification was more toxic than pristine GO. |  |
| GO and reduced GO (rGO) (100 nm) | 20, 40, 60, 80, and 100 µg mL^-1^ | 24 hours | THP-1 cells | Cellular viability, proliferation, oxidative stress, mitochondrial membrane potential, ATP synthesis, antioxidants, apoptosis, DNA damage, and the inflammation response | Both GO and rGO caused dose-dependent loss of cell viability and proliferation, increased level of LDH, MMP, decreased level of ATP content, redox imbalance, mitochondria-mediated apoptosis, cell death due to oxidative stress, increased secretion of various cytokines and chemokines. Overall, the toxic response of rGO was more severe than GO for all endpoints. | (113) |
| GO nanoplatelets (GONPs) and reduced GONPs (rGONPs) | GONP (5µg mL^-1^) or rGONP (50 µg mL^-1^ | 24 hours | THP-1 cells | Cell viability, ROS production, expression of genes related to the oxidative and inflammatory response, cellular uptake, endocytosis and phagocytosis, Rho/ROCK pathway, cytoskeleton analysis, differentiation of THP-1 cells into macrophage-like cells (THP-1a) | Both GO induced a dose-dependent loss in cell viability, an increase in ROS production, and a disruption of the F-actin cytoskeletons leading to the loss of the adherence ability of THP-1a and a reduction in the phagocytosis capability of THP-1a cells. GONP presented higher upregulation of HO-1 and SOD-2 expressions, and higher levels of IL-1β, TNF-α, IL-8, and MCP-1, compared to rGONP. rGONP exhibited a greater expression of NF-кB (p65), higher uptake and a higher decrease of Rho/ROCK expression than GONP. | (114) |
| Pristine graphene with 1% pluronic F108 | 20 µg mL^-1^ | 24 hours | Primary and immortalize (RAW264.7) macrophages | Quantification of cytokines and chemokines (IFNɤ, IL-1α, IL-2, IL-4, IL-5, IL-6, IL-10, IL-17, TNFα, and GM-CSF, MCP-1, MCP-3, RANTES, MIP-1α and MIP-1β). RT-PCR analysis of the mRNA levels of TNF-α, IL-1β, IL-6, iNOS and COX-2. Adhesion, phagocytosis and cytoskeleton assay. | Increased transcription and secretion of cytokines and chemokines, which is triggered by activation of the NF-kB signaling pathway; The cytokines and chemokines secreted by graphene-exposed macrophages further impaired the morphology of naïve macrophages by affect the actin structures and podosomes expansion, decreasing the adhesion and phagocytosis. | (140) |
| Pristine graphene with 1% pluronic F108 (500-1000nm) | 20 µg mL^-1^ | 12, 24 and 48 hours | Murine RAW 264.7 macrophages | Cell viability, ROS production, MMP, apoptosis, expression of proteins (Phospho-p38 MAPKinase (P-p38), p38 MAPKinase (p38), Phospho-JNK (P-JNK), JNK, Phospho-ERK (P-ERK), ERK, Phospho- Smad2, Smad2, Bim, Bax, caspase 3, Bcl-2, PARP and β-actin) and genes (TNF-α, TGF-β TGF-β receptor I, TGF-β receptor II, Smad2, Smad3, Smad4, Smad7, β-Actin) | Loss of cell viability at highest concentration (100 µg/mL); induction of intracellular ROS generation, depletion of MMP and apoptosis, all in a time- and dose-dependent way; activation of the mitochondrial pathways: MAPKs (JNK, ERK and p38) as well as the TGF- β-related signaling pathways. | (141) |
| Graphene nanoplatelets (1-10 layers) | 1, 5 and 10 μg cm^2^ | 24 hours | THP-1 macrophages | Phagocytosis, cytokine release and the involvement of the NALP3 inflammasome. | Frustrated phagocytosis, loss of membrane integrity at higher concentration, increase in cytokines expression, and activation of the NALP3 inflammasome. | (116) |
|  | pharyngeal aspiration: 50 μg per mouse. intrapleural injection: 5 μg per mouse | 24 hours | C57BL/6 strain mice | BAL cells analysis, Histological sections of lungs. Pleural space lavage: total and differential cell count, histological examination of the parietal pleura. | BAL and pleural lavage showed an increased number of polymorphonuclear leucocytes (neutrophils and eosinophils); and an increase int the levels of cytokines. Histological analysis: presence of granulomatous lesions in the bronchiole lumen and near the alveolar region; presence of histiocytic aggregates along the mesothelium. |  |
| Graphene nanoplatelets (~10 layers; particle size ~ 2 µm; thickness ~3–4 nm) | Intratracheal instillation: 1.25, 2.5 and 5 mg kg^-1^ | 90 days | ICR mice | Blood and BAL analysis: concentrations of pro-inflammatory cytokines (IL-1β, TNF-α, IL-6, IL-2, Th1-type cy- tokines, Th2-type cytokines) and chemokines (MIP)-1α, MCP-1, and GM-CSF in BAL fluids and immunoglobulins (Ig, IgE, IgG, and IgM) in serum. Expression of genes encoding actin family cytoskeletal proteins, calcium-binding proteins, and natriuretic-related genes. Histopathological analysis of lung. | BAL: increased number of lymphocytes, GNP-engulfed macrophages and apoptotic cells; general increase in cytokine and chemokine secretion; blood: increased number of macrophages and neutrophils, and elevated production of IgG, IgM and IgA. Gene expression: elevated expression of gens related to actin family cytoskeletal proteins and calcium-binding proteins; and alteration of natriuretic-related genes expression. Histopathological analysis: presence of GNP-engulfed macrophages without pathological lesion | (117) |
| Single- and multi-layered GO (SLGO and MLGO) in the presence or absence of Pluronic F-127 | 10, 20, 40, 80 and 100 μg mL^-1^ | 6 hours | THP-1 cells | Cell viability, membrane integrity, cell morphology levels of cytokine and ROS production, phagocytosis, and cytometric apoptosis. | SLGO induced ROS and IL-1β production, necrosis, and apoptosis to a lesser extent than MLGO. However, SLGO induced higher membrane damage and decrease in cell viability. | (125) |
|  | Iv: 10 mg kg^-1^ | 24 hours (acute toxicity) or 10 days (chronic toxicity) | Mice | Histological analysis of lung and kidney: immunohistochemistry (IHC) for MCP-1 and TGF-β. | Both SLGO and MLGO induced acute and chronic damage to the lung and kidney in the presence or absence of Pluronic F-127. |  |
| GO-PEG with mean thickness of 1.1 nm and lateral dimension ranged from 20 to 80 nm | It: 25 mg/kg | 28 days | Balb/c mice: Age: 6 - 8 weeks; Weight: 18 - 22g | Blood circulation test; Hematologic and Biochemical marker analysis; Histopathological evaluation: trace element biodistribution observation in heart, liver spleen, lung, kidney and lymph. | Blood exposure to GO under the maximum safe starting dose caused accidental death in 1/5 *Macaca fascicularis* and 7/221 mice, while remains general amenable in others. Elevated levels of immunoglobulin E and severe lung injury were found in dead animals, suggesting the GO-induced acute anaphylactic reactions. | (142) |
|  | 4 mg/kg | 90 days | *Macaca fascicularis*:Age; 4 - 5 years;Weight: 4- 5 kg |  |  |  |
| Graphene oxide – silver nanoparticles hybrid material (GOAg) | 5, 10 and 25 mgmL^-1^ | 24 hours | J774 and primary murine macrophages | Cell viability, apoptosis/necrosis, mitochondrial depolarization, lipid peroxidation, cytokines release (IL-1β, TNF-α and IL-10), ratio between CD80 and CD206 macrophage populations and NO production. | GOAg induced a dose-dependent mitochondrial depolarization, apoptosis, and lipid peroxidation to J774 macrophages. However, no effects were observed on cytokines release, macrophages polarization towards M1and NO production. | (143) |
| Bimetallic oxide FeWOx -PEG nanosheet (FeWOx-PEG) | 0-200 μg mL^-1^ | 24 hours | 4T1 and CT26 cells | Cell viability, internalization, ROS generation. | No significant toxicity was observed, however FeWOx-PEG could internalize via cell endocytosis and efficiently active OH generation and GSH depletion. | (115) |
|  | Toxicity: 10 mg kg^-1^  Biodistribution: 120 mg kg^-1^ |  | BALB/c mice | Body weight, histological analysis, blood chemistry, cytokines secretion (IL-6, IL-12 and TNFα) and biodistribution. | No significant differences in blood chemistry were observed for FeWOx-PEG treated mice. Also, H&E staining and histology analysis showed no obvious tissue damages and adverse effects and no significative body weight changes. However, FeWOx-PEG induce strong immune responses, showed by the increase levels of IL-6, IL-12 and TNFα. Biodistribution analyses showed that the material could accumulate in liver and spleen, however, it was observed a decrease concentration after 7 and 14 days indicating the biodegradable and clearable behavior of FeWO_X_ -PEG nanosheets. |  |
| FePSe_3_@APP@CCM | 0-160 μg mL^-1^ | Viability: 6 hours  Cytokine secretion: 48 hours | PBMC, CT26 and RAW-264.7 cells | Viability and cytokines secretion (IL-10, IL-12 and IFN- γ) | No obvious cytotoxicity was caused by the nanomaterial However, taken together, upon NIR laser irradiation, FePSe_3_@APP@CCM matured and activated immature DCs, enhanced the secretion of IFN-𝛾 and IL-12, and decreased the expression and the consequent inhibitory eﬀect of IL-10 on T cells, resulting in the enhanced immunity of T cells for killing CT26 cancer cells in the coculture system. | (144) |
|  | 10 mg kg ^−1^ | 25 days | C57BL/6J mice | Body weight, blood biochemical parameters (ALT, AST, BUN, CRE, LDH and PLT), histological analysis and cytokines secretion. | No obvious abnormality, inﬂammation and exudation or other pathological lesions were observed. Also, it was observed the increased expression of DC-secreted cytokines, including IFN-𝛾 and IL-12, while the level of IL-10 was found to be decreased |  |
| Ferrimagnetic vortex-domain iron oxide nanoring and graphene oxide (FVIOs-GO) hybrid nanoparticle | 50 or 75 μg mL^-1^ Fe | 8 and 24 hours | 4T1 breast cancer cell and RAW264.7 | Cell viability, uptake, apoptosis/necrosis, ROS generation, macrophages polarization. | Increased ROS generation and macrophage polarization to pro-inflammatory M1 phenotypes. | (145) |
|  | Iv: 3 mg kg^-1^ | 24 days | Balb/c mice Subcutaneous 4T1 Breast Tumor Model | Measurement of tumor width and length for 24 days. | Control group exhibited a rapid increase in the tumor volume, while FVIOs-GO group had tumor growth inhibition by 97.1%. |  |
| Borophene nanosheets (B NSs), graphene nanosheets (GR NSs) and phosphorene nanosheets (BP NSs) | Viability: 60, 80 and 100 μg mL^-1^  Membrane damage: 100 μg mL^-1^  Uptake: 200 μg mL^-1^ | Viability: 24 hours  Uptake: 6 hours | dTHP-1 and SC cells | Cell viability, membrane damage, cell uptake, intracellular localization, inflammatory cytokines secretion (IL-1β, IL-6, IL-8, IFN- γ and TNFα). | Corona coated 2D monoelemental nanosheets decreases cytotoxicity and cell membrane damage. For B NSs it was observed an increase in cellular uptake when the material was coronated, therefore corona may promote phagocytosis. Protein corona also stimulates the secretion of inflammatory cytokines. GR NSs and B NSs had immunoregulation behaviors only in the presence of plasma corona, while BP NSs had stronger immunoregulation behavior regardless of the absence and presence of corona. | (131) |
| Aggregated MoS_2_ and 2D MoS_2 (_exfoliated by lithiation or dispersed by Pluronic F87) | 6.25–50 μg mL^-1^ | 24 hours | THP-1 and BEAS-2B cells | Measurement of IL-8, TNF-α, and IL-1β levels | Aggregated MoS_2_ induced significant increases in IL-8, TNF-α, and IL-1β production, while there were significantly less effects of 2D MoS_2_ on cytokine and chemokine production. | (126) |
|  | 2 mg kg^-1^ | 40h and 21 days | C57Bl/6 mice | BALF and lung tissue were collected for measurement of LIX, MCP-1, IL-6, TGF-β1, and PDGF-AA levels and performance of Hematoxylin and Eosin (H&E) or Masson’s trichrome staining. | Aggregated MoS_2_ induced robust increasing in LIX, MCP-1 and IL-6 responses along with neutrophilic exudation into the BALF; while 2D MoS_2_ did not trigger cytokine or chemokine production in the lung. Histopathological changes were observed with aggregated MoS_2_ inducing focal areas of inflammation around small airways, while 2D MoS_2_ had little or no effect. |  |
| Exfoliated pristine and covalently functionalized MoS_2_ | 1, 10, 25, 50, 75 and 100 μg mL^-1^ | 24 hours | Raw-264.7 and human monocyte-derived macrophages | Cell viability, CD86 expression and secretion of TNFα and IL6. | Cell viability was reduced only at high concentration; no variation of CD86 levels in both RAW 264.7 cells and human monocyte-derived macrophages was registered; no increase in cytokine secretion was observed for both cell lines. | (119) |
| Pristine MoS_2_ and PEGylated MoS_2_ | 10 μg ml^-1^ | 24 hours | Primary mouse macrophages | Cytokine secretion (IL-6, IL-10, MCP-1, IFN-γ, TNF-α and IL-12). | Both materials significantly increased the secretion of cytokines such as IL-6, IL-12, TNF-α, IFN-γ and MCP-1. Interestingly, MoS_2_-PEG was found to elicit stronger cytokine secretion than the pristine MoS_2_, particularly involving IL-6, TNF-α, IFN-γ, and MCP-1. | (124) |
| MoS_2_ alone, MoS_2_–PEG or MoS_2_–PEG–CpG | 0, 5, 10, 20, 30, 40 and  50 μg mL^-1^ | 48 hours | RAW-264.7 cells and 4T1 cells | Cell viability, Cytokine release (TNF-α and IL-6), | MoS_2_ alone, MoS_2_–PEG or CpG alone had no effect on cytokine release while the MoS_2_–PEG–CpG significantly elevate the cytokine level. MoS_2_–PEG–CpG could elevate the expression of CD86 & CD80 and the percentage of matured DCs (CD80+ CD86+ DCs) was remarkably raised to 79.8% when combined with NIR irradiation. | (146) |
| Protein coated with different proteins (HSA, Tf, Fg and IgG) MoS_2_ NSs | 500 μg mL^-1^ | 12 and 24 hours | THP-1 cells | Cellular viability, cellular uptake and cytokine release. | Protein coated MoS_2_ NSs increase viability and decrease cytoplasmic membrane damage comparing with MoS_2_ NSs. Also, the presence of a protein corona decreased the secretion of cytokines. Among the four NSs the IgG coated MoS_2_ NSs enhanced uptake and cause more inflammatory cytokines. | (31) |
| MoS_2_ nanosheets (100 and 500 nm) | 0 – 128 μg ml^−1^ | 48 hours | DC cells | Cell viability, apoptosis, ROS generation, expression of CD40, CD80, CD86 and CCR7, secretion of proinflammatory cytokines (IL-12p70, IL6, IL-1β and TNF-α, DC homing ability. | Overall, there were no significant differences in cytotoxicity assays, however hight doses could promote DC maturation as observed by the expression of CD40, CD80 and CD86 and enhanced secretion of IL-6 and TNF- α. Also, MSNs upregulate ROS generation in DCs, further promoting cytoskeletal rearrangement and promoting the local lymphoid homing ability of DCs. | (147) |
| Black phosphorus nanosheet (BPNSs) and black phosphorus quantum dot (BPQDs) (~300nm) | 100, 50, 25, 12.5 μg ml^−1^ | 48 hours | H1299, L0-2, 293T, THP-1 cell line and SC human macrophages | Cell viability, cellular uptake (1, 3 or 6h), intracellular localization, ROS generation, cytokines release (IL-1 β, IL-6, IL-8, IL-9, IL-10, IFN- γ), NO and TNF- α generation. | A reduction of cytotoxicity was observed when BPNSs and BPQDs were coated with protein corona reduced. However, the corona facilitated the BP internalization and induced an increase in inflammatory cytokines and in ROS generation. Also, an induction of NO and TNF- α production were provoked by BP and corona coated BP. | (129) |
| Black phosphorus nanosheet (128 nm) | 15 μg.ml^-1^ | 24 hours | 4T1, F10, CT26 and Raw-264.7 cell lines | Cell morphology, cell expression diﬀerences, expression of the surface marker CD80 using flow cytometry, proteomic analysis, western blot analysis and immunofluorescence to analyze, expression of IL-10 (M2-related marker) and TNF- α (M1-related marker). | Corona coated black phosphorus nanosheet increase the expression of calcium signaling pathways and interact with STIM2 protein facilitating Ca^2+^ influx promoting macrophage polarization. | (130) |
| Few-layer two-dimensional black phosphorous (2D BP) | 10 to 500 μg.ml^-1^ | 24 hours (acute toxicity) or 21 days (chronic toxicity) | SAOS-2, HOb, L929 and hMSC cell lines | Cell viability and proliferation, ROS production, immunofluorescence to analyze cell morphology, inﬂammatory marker expression tested by LPS to analyzed cytokine generation (IL-10 and IL-6). | Black phosphorus did not show cytotoxicity on human mesenchymal stem cells and inhibits the metabolic activity of SAOS-2 cell line while inducing both proliferation and osteogenic differentiation in HOb cell and mesenchymal stem cells. Also, the presence of BP inhibits the ALP (an early marker of osteogenesis) expression in SAOS-2 cells and induces antiproliferative and apoptotic eﬀects by increasing the production of ROS on SAOS-2 cells. Besides, increase the inflammatory cytokine generation but inhibits proinflammatory mediators for the co-culture of SAOS-2 and HOb. | (148) |
| Black Phosphorus nanoflakes functionalized with TGF-β inhibitor and neutrophil membrane (NG/BP-PEI-LY) | 20 μg mL^-1^ | 24 hours (*in vitro*) | 4T1 and HUVEC cell line | Cell viability, ROS production, apoptosis, cytokine generation (IL-6 and TNF-α) | NG/BP-PEI-LY induced acute inflammatory responses, cause a decrease in viability, and increase apoptosis and ROS production when laser irradiated. | (121) |
|  |  | 72 hours (*in vivo*) | BALB/c mice | Mice NIR fluorescent imaging, immunofluorescent staining of CD31 (red) and ICAM-1 (green). | Besides, when laser irradiated increased the ICAM-1 expression, enhancing intracellular delivery by adhesion molecule mediated targeting. |  |
| Black Phosphorus nanosheet (BPNS) and Black Phosphorus nanocomposite (BPCP) modified with PEG and OD CpG or CpG-Cy5.5 | Up to 100 μg mL^-1^ | 24 hours | 4T1, RAW-264.7 and Hep62 | Cell viability, necroptosis, protein expression, cytokine generation (IL-6 and TNF-α) and hemocytolysis. | No obvious citotoxicity was observed, also no significant hemolysis. For BPTT treatments it was observed that necroptosis play an important role, mediating death process in cancer cells. These results were confirmed by the expression of necroptosis-related proteins, where it was observed a significantly expression of RIP1 and RIP3. Caspase-8 and Caspase-3 levels were not significantly changed. | (149) |
|  | 2 mg/kg | Up to 16 days | BALB/c mice | Biodistribution, expression of immune factors (FOXP3, IL-2, TNF- α and INF- γ), histological analysis, hematological toxicity. | No body weight loss and no systemic toxicity were observed. Also, no tissue damage and blood physiological indicators were within normal range. After BPTT treatments the immune responses were activated as observed by detection of T lymphocytes and various immune cytokines. |  |
| DSPE-PEG coated TiO_2_ nanosheet (92.5 nm) | 1 mg mL^-1^ | 30 days | C57 mice | Body weight, biodistribution, immunogenicity, hematological toxicity, liver and spleen histopathology, oxidative stress response. | DSPE-PEG coated TiO_2_ nanosheet cause a decrease in body weight after 14 to 30 days of the injection, also, it was observed a that the particles were accumulated in liver and cause liver toxicity by inducing oxidative stress. Besides, an obvious decrease in HTC and significant increase in MCH and MCHC indicate that the particles may induce blood system damage. | (150) |
| Two-Dimensional Core − Shell  MXene@Gold Nanocomposites | *In vitro*: 3.1 to 100 μg mL^-1^ | 24 hours | 4T1 cell line | Cell viability, immunohistochemistry and immunofluorescence staining. | Overall, the particle did not show apparent cytotoxicity, and no toxic side effect was observed in mice after 30 days of injection. | (151) |
|  | *In vivo*: 20 mg kg^-1^ | 30 days | Balb/c mice | Body weight and biodistribution. | No heigh loss and no notable abnormality on major organs were observed. |  |
| 2D titanium nanosheets (TiNS) and polyethylene glycol coated titanium nanosheets (TiNS-PEG) | *In vitro*: 10-100 ppm | 4 hours | B16 cell line, J774A.1 cell line and SMMC-7721. | Cell viability. | TiNS and TiNS-PEG did not significantly affect cell viability. | (120) |
|  | *In vivo*: 5 mg kg^-1^ | 19 days | Balb/c mice | Histopathology, body weight, biodistribution and hematological toxicity. | Any significant differences on mice body weight, no histological abnormalities, and no impact on hematological parameters, indicating no inflammation and other negative impact on blood and organs was observed. |  |
| PEGylated molybdenum dichalcogenides (MoS_2_-PEG), tungsten dichalcogenides (WS_2_-PEG) and titanium dichalcogenides (TiS_2_-PEG) nanosheets | *In vitro*: 25 – 200 μg mL^-1^ | 24 hours | RAW-264.7, 4T1 and 293T. | Cell viability and ROS generation. | No significant *in vitro* cytotoxicity was observed for all the three types of PEG functionalized TMDCs. | (152) |
|  | *In vivo*: 10 mg kg^-1^ | up to 60 days post injection | Balb/c mice. | biodistribution, hematological toxicity, biochemical parameters (ALP, ALT, AST and BUN) and histopathology. | The materials show dominate accumulation in reticuloendothelial systems (RES) such as liver and spleen after intravenous injection. Also, no significant results were observed for the analysed biochemical and haematological parameters and no obvious sign of abnormality, such as inflammation, was noticed in all examined major organs. |  |
| Two-dimensional polyethylene glycol modified TiS_2_ nanosheets (TiS_2_-PEG) | *In vitro*: 0.0015 - 0.1 mg ml^-1^ | 24 hours | 4T1 cell and | Cell viability | No significant cytotoxicity of TiS_2_-PEG was observed. | (153) |
|  | *In vivo*: 20 mg kg^-1^ | 60 days | Balb/c mice | *In vivo* toxicity, histopathology. | No histological abnormalities and no obvious toxicity to Balb/c mice was observed. |  |
| BSA coated 2D silicene nanosheets (SNSs-BSA) | *In vitro*: 12.5 - 200 μg mL^-1^ | 24 hours | 4T1 and U87 cell lines | Cell viability | SNSs-BSA exhibit insignificant effect on cell viability of either 4T1 or U87 cancer cells. | (127) |
|  | *In vivo*: 20 mg kg^-1^ | 4 weeks | Kunming mice and Balb/c mice | Body weight, histopathology, hematological toxicity, biochemical parameters (ALT, AST, ALP, urea, CREA, and UA). | In a four-week duration, the mice present no significant abnormality, body weight differences, and no significant behavioral alterations. The histological observations of major organs showed no significant acute pathological toxicity. Furthermore, haematological parameters showed no obvious sign of abnormalitys indicating that the SNSs-BSA induce negligible renal and hepatic toxicity in mice model. |  |
| Poly(vinylpyrrolidone)-encapsulated Bi_2_Se_3_ nanosheets (diameter 31.4 nm and thickness 1.7 nm) | *In vitro*: 5 - 200 ppm | 48 hours | MCF7 cell line | Cell viability | It was not observed any cytotoxicity effects caused by Bi_2_Se_3_ nanosheets. | (154) |
|  | *In vivo*: 27 – 1168 mg kg^-1^ | 14 days | Balb/c mice | *in vivo* toxicity and biodistribution. | At the dose of 750 or less no mice mortality nor any reaction was observed. The nanomaterial mainly accumulated in liver, spleen and kidney, however, the concentration decreases with time. |  |
| Pd nanosheets  (diameter ranging from 5 to 80 nm) | *In vitro*: up to 100 μg mL^-1^ | 24 hours | NIH-3T3, 4T1, Raw-264.7, QSG-7701 and QGY-7703 cell lines | Cell viability, mitochondrial membrane depolarization and ROS generation. | Pd nanosheets have no effect on cell viability, apoptosis, ROS generation, or mitochondrial depolarization. | (112) |
|  | *In vivo*: 10 mg kg^-1^ | 30 days | Balb/c mice | Biodistribution, blood chemistry and hematology analysis and histopathology. | The *in vivo* results show that the particle is primarily trapped by reticuloendothelial system (RES). Also, no significant hepatotoxicity was induced by Pd nanosheets of different sizes. The activity of ALP, ALT, AST and BUN observed was within normal range and no apparent histopathological abnormalities or lesions were observed in any major organ. |  |
| PEGylated ultrathin boron nanosheets (B-PEG NSs) | 25 to 500 µg mL^−1^ | 48 hours | HeLa, PC3, MCF7, and A549 | Cell viability, ROS generation. | No significant cytotoxicity was observed for B-PEG NSs. However, when exposed to an 808 nm NIR laser (1 Wcm^−2^) for 5 min it was notices a strong concentration-dependent cytotoxicity. Also, when the B-PEG NSs were combined with DOX and NIR laser irradiation, over 95% of the cells died at a DOX concentration of 100 µg mL^−1^. | (80) |
|  | 5.3 mg kg^-1^ | 24 hours | Mice | Body weight, histopathology, hematological toxicity (HGB, WBC, RBC, MCV, MCHC, PLT, MCH, HCT, Cr, NEU, LYM, MPV), biochemical parameters (ALP, AST, BUN and ALT) and cytokine generation (TNF-α, IL-6, IFN-γ, and IL-12+P40) | No obvious side effects were noted, also the levels of TNF-α, IL-6, IFN-γ, and IL-12+P40 were similar to those in the PBS control group indicating that B-PEG NSs did not induce obvious cytokine response. Compared with the control group, there is no statistically significant difference of the NSs-treated groups with PBS-treated groups in all the parameters, no obvious induction on cytokine response, no change in biochemical parameter and no hematological toxicity, therefore, B-PEG NSs do not cause obvious infection and inflammation in the treated mice. Moreover, no noticeable signal of inflammation or tissue damage was observed in major organs. |  |

^1^Ip: intraperitoneal; ^2^Lung: Oropharyngeal Aspiration; ^3^It: intratail. Initials: poly-(ethylene glycol)-functionalized GO (GO-PEG); flavin mononucleotide-stabilized pristine graphene (PG-FMN); aminated GO (GO-NH2); poly(acrylamide)-functionalized GO (GO-PAM); poly(acrylic acid)-functionalized GO (GO-PAA); polyethylene glycol (PEG); N-(carbonyl-methoxypolyethyleneglycol 5000)-1,2-distearoyl-sn-glycero-3-phosphoethanolamine (DSPE-PEG); human serum albumin (HSA); transferrin (Tf), fibrinogen (Fg), and immunoglobulin G (IgG). Nanosheets (NSs); aspartate aminotransferase (ALP); alanine aminotransferase (ALT); lactate dehydrogenase (LDH); Blood urea nitrogen (BUN); Creatinine (CRE); lactate dehydrogenase; platelet (PLT); nitric oxide (NO); immunohistochemistry (IHC); Nuclear magnetic resonance spectroscopy (NMR); tricarboxylic acid cycle (TCA); Polyvinyl chloride (PVP); lipopolysaccharide (LPS); Rho-associated protein kinase (Rho/ROCK); red blood cells (RBC); white blood cells (WBC); mitochondrial membrane potential (MMP); mitogen‑activated protein kinase (MAPKs); extracellular signal-regulated kinase (ERK); c-Jun N-terminal kinase (JNK); Glutathione (GSH); bronchoalveolar lavage fluid (BALF); lymphocytes (LYM); Mean platelets volume (MPV); hematocrit count (HTC); hemoglobin (HGB); mean volume cell (MCV); mean cell hemoglobin (MCH); MCH concentration (MCHC); red blood cell (RBC); neutrophil count (NEU); doxorubicin (DOX); near infrared light (NIR); uric acid (UA); cytosine–phosphate–guanine (CpG); black phosphorus based photothermal therapy (BPTT); transition metal dichalcogenides (TMDC). Cell lines: mouse BALB/c monocyte macrophage (j774.a1); human monocyte cell line isolated from the peripheral blood of a male infant with acute monocytic leukemia (THP-1); peripheral blood mononuclear cell (PBMC); human T lymphocyte cells (Jurkat); mouse monocyte macrophage (RAW-264.7); dendritic cells (DC); bronchoalveolar lavage cells (BAL); mouse mammary adenocarcinoma cell line (4T1); murine colorectal carcinoma cell line (CT26); human peripheral blood mononuclear cells (PBMCs); human non-small cell lung carcinoma cell line (H1299); immortal hepatic cell line (L0-2); stable clone derivative of the human embryonic kidney (HEK) 293 cell line (293T); human macrophages from peripheral blood (SC); mouse melanoma cells (F10); human primary osteogenic sarcoma cells (SAOS-2); human adult osteoblasts (HOb); murine ﬁbroblast cell line (L929); human mesenchymal stem cell (hMSC); human umbilical vein endothelial cell (HUVEC); human liver cancer cell line (Hep62); human hepatocarcinoma cell line (SMMC-7721); human glioblastoma cell line (U87); human hepatocyte line (QSG-7701); human hepatocellular carcinoma (QGY-7703); human cervix epitheloid carcinoma cells (HeLa); human Caucasian prostate adenocarcinoma (PC3); human Caucasian breast adenocarcinoma (MCF7); human Caucasian lung carcinoma (A549).
